# Male-derived PBP4 is essential for sperm competition by mediating sperm motility in moths

**DOI:** 10.1073/pnas.2510155122

**Published:** 2025-10-28

**Authors:** Yu He, Qi Yan, Jing-Hao Hou, Ying Li, Zhi-Qiang Wei, Jin-Meng Guo, Nai-Yong Liu, Markus Knaden, Bill S. Hansson, Shuang-Lin Dong, Jin Zhang

**Affiliations:** ^a^State Key Laboratory of Agricultural and Forestry Biosecurity, College of Plant Protection, Nanjing Agricultural University, Nanjing 210095, China; ^b^Key Laboratory of Integrated Management of Crop Disease and Pests (Ministry of Education), Nanjing Agricultural University, Nanjing 210095, China; ^c^Key Laboratory of Forest Disaster Warning and Control of Yunnan Province, Southwest Forestry University, Kunming 650224, China; ^d^Department of Evolutionary Neuroethology, Max Planck Institute for Chemical Ecology, Jena 07745, Germany; ^e^Key Laboratory of Soybean Disease and Pest Control (Ministry of Agriculture and Rural Affairs), Nanjing Agricultural University, Nanjing 210095, China

**Keywords:** pheromone-binding protein, sperm motility, sperm competition, *Spodoptera exigua*, PBP-OR pathway

## Abstract

Male moths rely on antennal olfactory sensilla to detect female-emitted sex pheromones during mate localization, a process critically mediated by pheromone-binding proteins (PBPs) that facilitate ligand transport to dendritic membrane-bound pheromone receptors. Intriguingly, a divergent PBP paralog (PBP4) in noctuid moths has been identified exhibiting predominant expression in the male reproductive system, though its functional significance remained elusive. In this study with *Spodoptera exigua*, we demonstrated that PBP4 is specifically expressed in male accessory glands (MAGs) and transferred to females during copulation. CRISPR-mediated PBP4 knockout (PBP4^−/−^) significantly reduced male reproductive success via impaired sperm competition, evidenced by diminished offspring sired. Transcriptomic and qPCR analyses revealed expressional correlation between PBP4 and the odorant receptor coreceptor (Orco), and similar sperm competition deficits were observed in Orco^−/−^ males. Furthermore, both mutants exhibited compromised sperm motility and reduced sperm transfer from spermatophores to spermathecae. In vitro sperm motility assays showed that a specific agonist of Orco, VUAA1, robustly enhanced sperm motility in an Orco-dependent manner. Analyses of four additional moth species, including noctuids and non-noctuids, revealed a similar specific expression in the male reproductive tracts in all of them. Our findings unveil a noncanonical role for PBP4 in reproductive fitness and evolutionary conservation of the PBP-OR signaling pathway.

## Significance

This study reveals a role of pheromone-binding protein 4 (PBP4) in moth reproduction. Unlike its ancestral olfactory function, PBP4 is expressed specifically in male accessory glands (MAGs), transferred to females during mating, and enhances sperm competition by promoting sperm motility and transfer via a conserved PBP-OR pathway. The pathway, previously linked to antennal pheromone detection, also regulates sperm competitiveness, linking chemical communication and reproductive biology. These findings redefine PBPs as multifunctional players in both chemical ecology and reproduction, offering insights for pest control strategies targeting fertility.

Insects have developed highly efficient and precise olfactory systems for locating hosts and mates (1). Male moths possess a pair of highly developed antennae, by which they can detect conspecific sex pheromones, and accordingly locate female partners for mating with high efficiency (2). Tens of thousands of sensilla are distributed on the antennae, of which most house olfactory sensory neurons (OSNs) sensitive to the sex pheromones, endowing the male moths with extremely high sensitivity in sex pheromone perception (3). The pheromone-binding proteins (PBPs) exist in high concentration in the sensillum lymph and play an indispensable role in recognizing and transporting the pheromone molecules across the aqueous sensillum lymph toward the pheromone receptors situated in the membrane of the OSNs (4).

In Lepidoptera, the number of PBPs ranges from one to five in a single species, but three PBPs are exclusively expressed in the antenna of noctuid species (5–12). The PBPs of Lepidoptera are categorized into four groups (PBP-A, B, C, and D) based on similarity of amino acid sequences (8). Being the typical nocturnal insects and largest family within Lepidoptera, noctuid species have attracted most studies regarding PBPs and their functions. Three PBP genes (*PBP1*, *PBP2*, and *PBP3*) are commonly expressed in the antennae of a noctuid species (13–16), forming three distinct subgroups (*PBP1*, *PBP2*, and *PBP3*, corresponding to PBP-D, A, and C in previous literature), presumably originating from an ancestral gene by duplication events (17). All three PBPs play roles in the perception of female sex pheromones, although with different contributions regarding the detection of different components of the pheromone blend (18–20). The first *PBP4* among the Noctuidae was identified in *Spodoptera litura (SlitPBP4)*, by analyzing the transcriptome data from the male reproductive system, displaying a high RNA expression in the male reproductive system but little in antennae. This distinct expression profile implies that the *PBP4* gene has a different function than perception of external sex pheromones, but rather in male reproductive physiology (21). Later, a *PBP4* ortholog was discovered in *Spodoptera frugiperda*, showing a similar expression profile as *SlitPBP4* (22). This expression profile explains why the *PBP4* gene in noctuid species was found much later than *PBP1-3*, as researchers mostly focused on the antennae and other olfactory tissues. Our phylogenetic analysis showed that the *PBP4* genes of Noctuidae species were clustered into a clade with PBP-Bs (*Bombyx mori PBP4*, *Manduca sexta PBP4* and *Danaus plexippus PBP1*) from three non-Noctuidae species (*SI Appendix*, Fig. S1). In addition, the amino acid sequence encoded by *PBP4* exhibited a notable differentiation to that of *PBP1-3*, with approximately 40 additional amino acids present at the C-terminus (21). However, the *PBP4* gene is closely located with the genes of *PBP1-3* on the same chromosome, suggesting a common ancestor of the four PBPs. Clarifying the function of *PBP4* in the male reproductive physiology thus holds the potential to unveil a function of moth PBPs.

In the present study of *Spodoptera exigua*, we first determined that PBP4 was exclusively expressed in the male accessory glands (MAGs) and was transferred to the female during mating. We then demonstrated that PBP4 plays a crucial role in sperm competition using PBP4 knockout males. To elucidate the mechanism, transcriptome analysis and qPCR verification suggested the involvement of the *Orco* gene in sperm competition, which was confirmed by mating experiments using *Orco* mutants. Further assays showed that knocking out either *PBP4* or *Orco* significantly decreased the number of sperm transferred from the spermatophore to the spermatheca. Finally, sperm motility assays demonstrated that crude extracts from WT male spermatophores significantly enhanced sperm motility compared to those from *PBP4*^−/−^ males, and that VUAA1 (an agonist of Orco) (23, 24) enhanced the motility of sperm from both wild-type and *PBP4*^−/−^ males but not from *Orco*^−/−^ males. Overall, we conclude that the moth PBP4 is essential for sperm competition by enhancing the sperm motility via the PBP-OR signaling pathway.

## Results

### PBP4 Protein Is Expressed in MAGs and Transferred to Females Via Seminal Fluid.

**Fig. 1.**
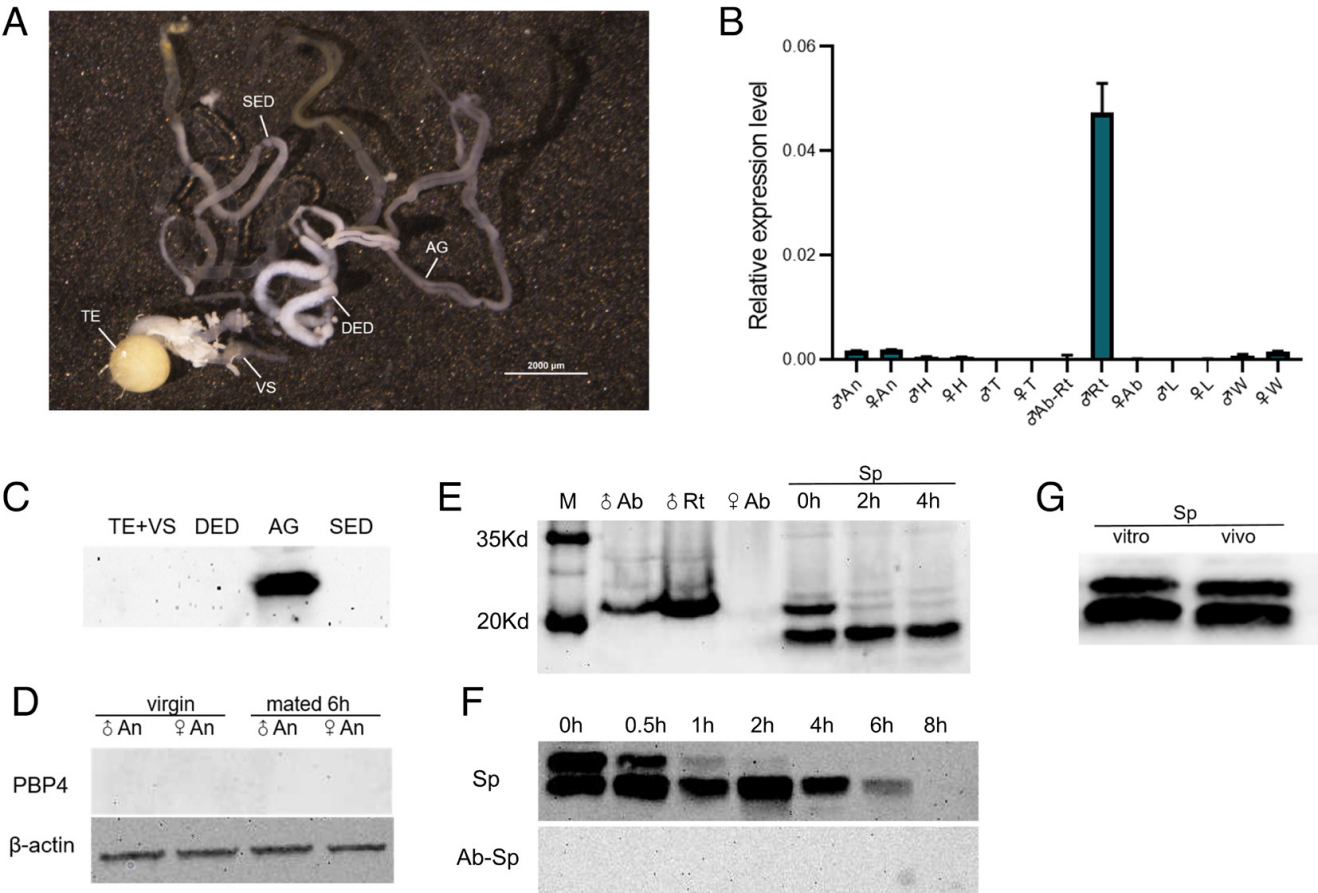
PBP4 protein is specifically expressed in the male accessory glands (MAGs) and transferred to females via mating. (*A*) Schematic of the male reproductive system of *S. exigua*. Abbreviations: TE, testis; VS, vesiculae seminales; DED, double ejaculation ductus; SED, single ejaculation ductus; AG, accessory glands. (*B*) qRT-PCR analysis of *SexiPBP4* mRNA levels in different tissues. Abbreviations: An, antennae; H, head; T, thorax; Ab, abdomen; Rt, reproductive tract; Ab-Rt: abdomen with the reproductive tract removed. L, leg; W, wing. (*C*) Western blot of PBP4 in various parts of the reproductive system of male moths. (*D*) Western blot of PBP4 in antennae of virgin and mated male and female moths. (*E*) Antisera against PBP4 recognize a predicted and a shorter molecular mass in spermatophore. Abbreviations: Sp, spermatophore. (*F*) PBP4-immunoreactive protein is hardly detectable in spermatophore at 8 h after mating and undetectable in residual abdomen. (*G*) PBP4 antiserum also detected the cleaved protein in the spermatophore produced by the in vitro mating method.

A previous study using qPCR showed that *PBP4* from *S. litura* was only slightly expressed in both male and female antennae, but highly expressed in the male reproductive system (21). In the present study with *S. exigua*, qPCR analysis showed a similar tissue expression pattern, i.e. a high expression level in the male reproductive system and low levels in antennae and wings (Fig. 1*B*). Western blot analysis further confirmed the high expression of PBP4 in MAGs (Fig. 1*C*). Notably, PBP4 was not detected in the antennae of either males or females, neither before nor after mating (Fig. 1*D*).

To determine whether the PBP4 is transmitted to females as a component of semen during mating, we examined the protein’s presence in the female abdomen and in the transferred spermatophore using western blot analysis. PBP4 was absent in the abdomen of virgin females, but was detected in the spermatophore postmating. In the spermatophore, two bands were observed (one at the expected molecular weight and the other at a lower weight). The intensity of the expected band diminished over time, suggesting that an enzymatic cleavage of the protein occurred (Fig. 1*E*). Further analysis showed that PBP4 did not transfer from the spermatophore into the female hemolymph. Instead, it was cleaved completely in about 1 h and degraded entirely in 8 h (Fig. 1*F*). Notably, the PBP4 protein in the spermatophore produced in vitro (without female participation) also underwent cleavage (Fig. 1*G*), indicating that a specific enzyme responsible for this cleavage was transmitted from the males together with PBP4. Thus, the PBP4 protein is synthesized in the MAGs, transferred to females during mating, and functions within the spermatophore.

### *PBP4* Plays a Crucial Role in Sperm Competition.

**Fig. 2.**
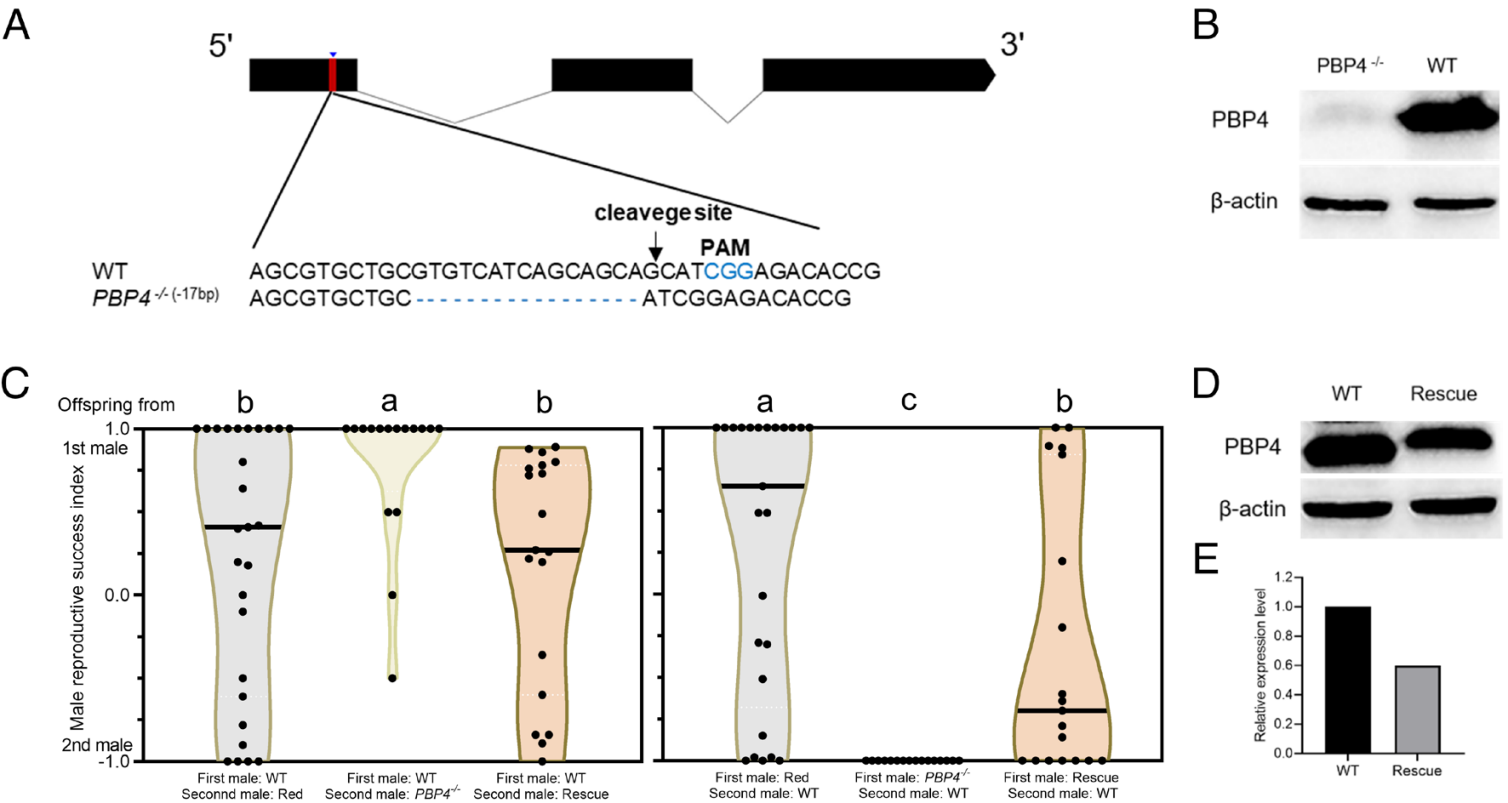
Mutation of *PBP4* damages sperm competition ability. (*A*) Schematic diagram of single-guide RNA (sgRNA) targeting site of *SexiPBP4*. The protospacer adjacent motif (PAM) sequence is in blue. The change in length is shown *Left* of each sequence (–, deletion). (*B*) Western blot of PBP4 in accessory glands of WT and *PBP4*^−/−^ males. (*C*) Violin plots represent male reproductive success index on the first day. Different letters indicate significant differences among groups (Welch and Brown–Forsythe one-way ANOVA with Tamhane T2 test, *P* < 0.05). (*D*) Western blot of PBP4 in accessory glands of WT and *PBP4* rescued males. (*E*) The relative PBP4 expression level in WT insects and rescued mutants.

To elucidate the function of PBP4, the recombinant PBP4 protein was expressed, and fluorescence competitive binding assays were performed with four sex pheromones of *S. exigua*, but no binding affinity was observed for any of the pheromones tested (*SI Appendix*, Fig. S2). Then, we generated a null mutation in the gene *PBP4* using CRISPR-Cas9 genome editing. Among various mutations, a 17-bp deletion was selected to establish a homozygous *PBP4* knockout strain (*PBP4*^−/−^) (Fig. 2*A*), which was predicted to produce a nonfunctional protein comprising only 44 amino acids. Western blot analysis confirmed the absence of PBP4 in MAGs of the knockout strain (Fig. 2*B*).

To determine the function of PBP4, we compared the reproductive parameters between females mated with WT and *PBP4*^−/−^ males. These parameters included oviposition capacity, egg hatchability, mating, and remating ability. None of these parameters showed significant differences between the two groups (*SI Appendix*, Table S3). Additionally, the sex pheromone content (at 2 to 3 h postmating) and the calling rhythm (on the second day) were comparable between females mated with *PBP4*^−/−^ and WT males (*SI Appendix*, Fig. S3 *A* and *B*).

Given the fact that *S. exigua* females, like many other insects, mate multiple times, we investigated the role of PBP4 in sperm utilization pattern. A *piggyBac*-based transgenic insect strain expressing red fluorescent protein was constructed to track sperm utilization (*SI Appendix*, Fig. S4 *A* and *B* and Table S4). Mating experiments were conducted, in which females mated with one male type on the first day and with another type on the second day. The number of offspring sired by each male was determined using a fluorescence stereomicroscope. Regardless of mating order, sperm from the first male was more likely to fertilize the female compared to that of the second male (Fig. 2*C* and *SI Appendix*, Fig. S4*E*), indicating a significant “first male precedence” pattern.

To determine whether *PBP4* plays a role in sperm competition, we examined the offspring genotypes of twice-mated WT females by DNA sequencing. In both mating orders, WT males sired a significantly higher number of larvae (Fig. 2*C* and *SI Appendix*, Fig. S4*E*), highlighting the essential role of *PBP4* in sperm competition. To rule out the potential effects of the female genetic background, the experiment was repeated with *PBP4*^−/−^ females, yielding similar results to WT females (*SI Appendix*, Fig. S4*F*). To further validate the results, a *PBP4* rescue strain was generated using a *piggyBac*-based transgenic vector, pBac[PBP4:his6] (*SI Appendix*, Fig. S4 *C* and *D* and Table S4). The expression level of PBP4 protein in the rescued strain was about 60% of WT strain (Fig. 2 *D* and *E*). As expected, when the rescued males mated as first partner, the number of offspring from the rescued males was significantly higher than that of *PBP4*^−/−^ males, but lower than that of WT males (Fig. 2*C* and *SI Appendix*, Fig. S4*E*); when rescued males mated as second partner, the number of offspring from the rescued males was significantly higher than that of *PBP4*^−/−^ males (Fig. 2*C*).

### Expression of *Orco* Is Correlated With That of *PBP4* and Contributes to Sperm Competition.

**Fig. 3.**
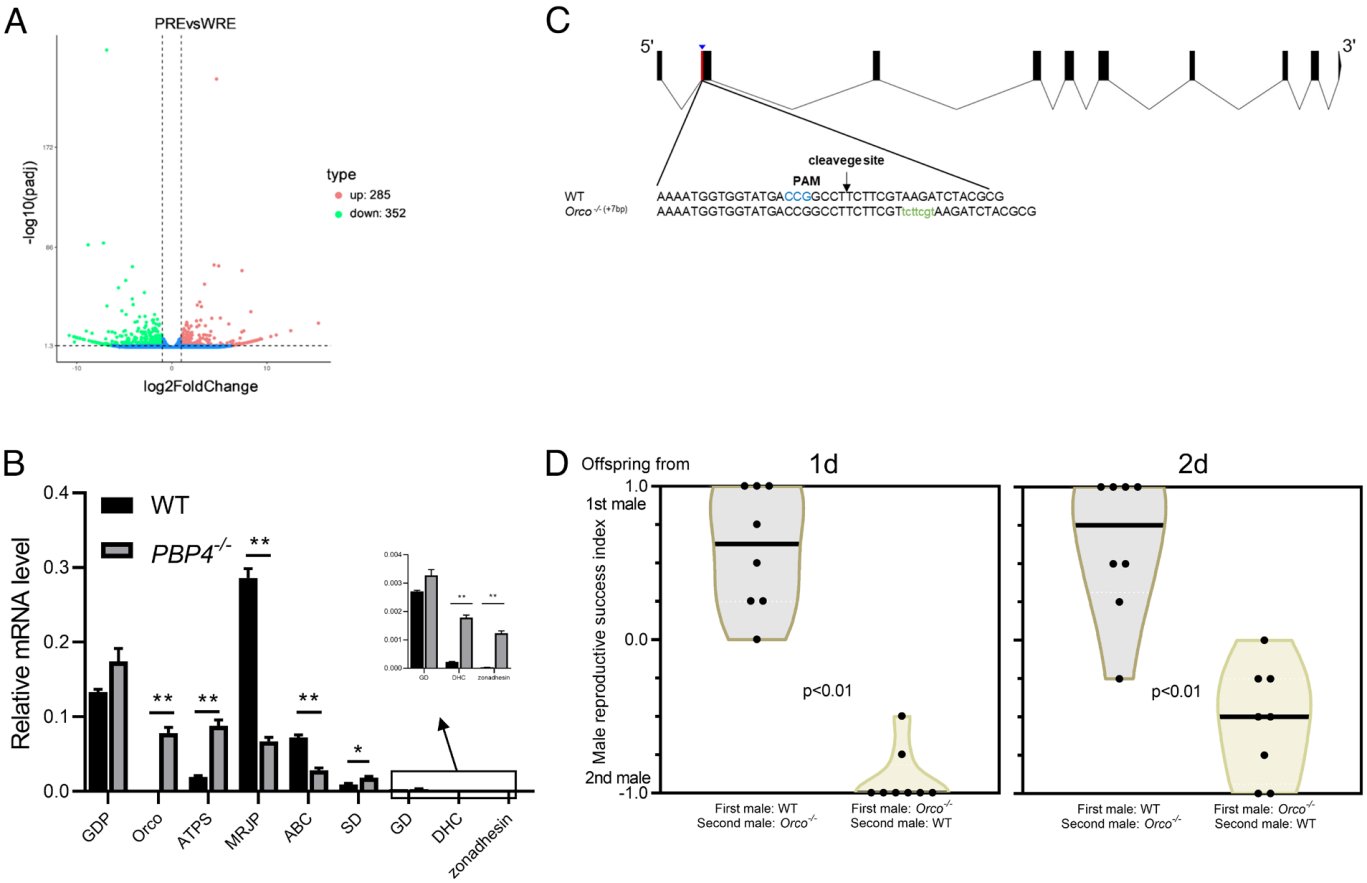
Mutation of *Orco* damages male sperm competition ability. (*A*) Transcriptome analysis of reproductive tract gene expression between WT and *PBP4*^−/−^ males. (*B*) qRT-PCR analysis of 9 candidate DEGs of male reproductive tract. GDP, glucose-induced degradation protein; ATPS, ATP-citrate synthase; MRJP, major royal jelly protein; ABC, ATP-binding cassette; SD, Sorbitol dehydrogenase; GD, Glucose dehydrogenase; DHC, dynein heavy chain. Error bar means SEM (n = 3). * indicate significant difference between the two groups (Student’s *t* test, *P < 0.05, **P < 0.01). (*C*) Schematic diagram of single-guide RNA (sgRNA) targeting site of *SexiOrco*. The PAM sequence is in blue. The change in length is shown *Left* of each sequence (+, insertion). (*D*) Violin plots represent male reproductive success index. The terms “1d” and “2d” denote the offspring produced on the first and second day, respectively, by females that mated twice. The difference of male success reproductive index after changing the order of mating was tested via the Mann–Whitney *U* test.

To explore the mechanism underlying PBP4 function, we conducted comparative transcriptome analyses on the reproductive tracts of WT and *PBP4*^−/−^ males, as well as the heads and abdomens of females mated with WT and *PBP4*^−/−^ males, respectively. In the male reproductive tracts, we identified 637 differentially expressed genes (DEGs), including 285 up-regulated and 352 down-regulated genes (Fig. 3*A*). Among these DEGs, nine DEGs were potentially involved in energy metabolism, sperm motility, and fertilization. Notably, the odorant receptor coreceptor (*Orco*) was significantly up-regulated by 167 times in the *PBP4*^−/−^ moths (*SI Appendix*, Table S6). In contrast, only 4, 54, and 28 DEGs were observed in heads and abdomens of females mated with WT and *PBP4*^−/−^ males at 2, 6, and 24 h postmating, respectively (*SI Appendix*, Fig. S5 *A*–*C*). Six DEGs showing the greatest differences at 6 h after mating were selected for qPCR validation (*SI Appendix*, Table S6), but none exhibited significant difference (*SI Appendix*, Fig. S5*D*). However, of the nine male reproductive tract DEGs tested by qPCR, six showed the same directional changes as in the transcriptome analyses (Fig. 3*B*), with *Orco* displaying the highest change (8,643 times). The correlated expression of PBP4 and Orco suggested that the two proteins function within the same pathway in the spermatophore, perhaps analogous to that in male antennae for sex pheromone perception.

Given the known roles of ORs and Orco in mammalian and insect sperm (25–27), we hypothesized that Orco may express in sperm and contribute to sperm activation in *S. exigua*. To verify this hypothesis, we generated an *Orco* knockout strain (*Orco^−/−^*) using CRISPR-Cas9 technology (Fig. 3*C*). Surprisingly, *Orco^−/−^* males were still capable of exhibiting sexual excitation toward and successfully mated with females, despite the loss of electrophysiological response to sex pheromones and plant volatiles (*SI Appendix*, Fig. S6*A*) and a notable decrease in mating rate (*SI Appendix*, Fig. S6*B*). There is no significant difference in egg number (*SI Appendix*, Fig. S6*C*) and egg hatchability (*SI Appendix*, Fig. S6*D*) between females mated with WT and *Orco^−/−^* males, indicating that *Orco* knockout had no clear effects on female’s fecundity. Interestingly, *Orco* knockout led to a 1.5-fold increase in *PBP4* expression in the male reproductive tracts (*SI Appendix*, Fig. S7*B*). Furthermore, the impact of *Orco* knockout on sperm competition was similar to that of *PBP4*^−/−^ males (Fig. 3*D*). Thus, both Orco and PBP4 were crucial for sperm competition, indicating the two genes function in a common pathway.

### Mutation of *PBP4* or *Orco* Reduces the Number of Sperm Transferred Into the Spermatheca.

**Fig. 4.**
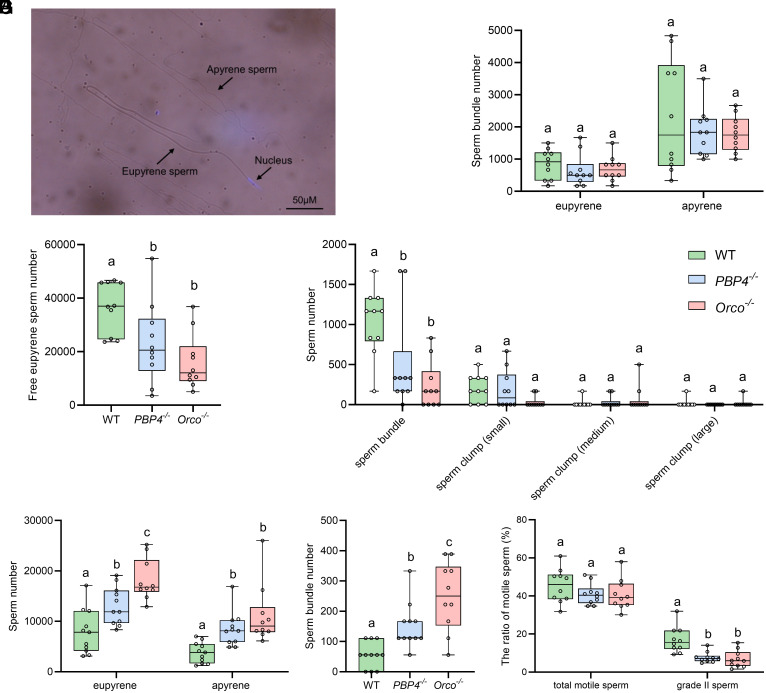
Decreased sperm motility of *PBP4*^−/−^ and *Orco^−/−^* males leads to a reduction of sperm transferred to the spermatheca. (*A*) Images of eupyrene and apyrene sperm. Nuclear staining was performed using DAPI. (*B*) The number of eupyrene and apyrene sperm bundles in spermatophore. (*C*) The number of free eupyrene sperm in spermatheca. (*D*) The number of sperm bundles and clumps in spermatheca. (*E*) The number of residual free eupyrene and apyrene sperm in spermatophore. (*F*) The number of residual sperm bundles in spermatophore. (*G*) The ratio of motile sperm of 3 strains. n = 10 to 11 (*B*–*G*). Boxplots depict median, upper, and lower quartiles. Different letters indicate significant differences among three insect strains (one-way ANOVA followed by the Tukey HSD test, *P* < 0.05).

The above-described results demonstrated that mutations of *PBP4* or *Orco* led to reduced sperm competition ability. To elucidate the mechanism, we first examined the quantity of sperm present in the spermatophore immediately after mating. In Lepidopteran insects, males produce two morphologically distinct types of sperm, eupyrene sperm (nucleate), and apyrene sperm (anuclear) (28), as also seen in *S. exigua* (Fig. 4*A*). Upon completion of mating, undissociated eupyrene sperm bundles (*SI Appendix*, Fig. S8*A*) and incompletely dissociated apyrene sperm bundles (*SI Appendix*, Fig. S8*B*) were observed in the spermatophore. However, there were no significant differences in the quantity of sperm bundles among the three insect strains (Fig. 4*B*), indicating that mutations in *PBP4* or *Orco* did not affect male ejaculation volume or the number of sperm entering into the spermatophore.

Following sperm activation, sperm exit the spermatophore and migrate to the spermatheca for storage (29). We thus investigated whether *PBP4* or *Orco* mutation affects sperm transfer into spermatheca. Unlike other moth species (30, 31), sperm transfer in *S. exigua* began at approximately 14 h postmating, with no discernible differences in the morphology of reproductive tracts among the three strains at this time point (*SI Appendix*, Fig. S9). By 16 h postcopulation, the majority of sperm had been transferred to the spermatheca (*SI Appendix*, Fig. S9), including free eupyrene sperm (*SI Appendix*, Fig. S8*C*), sperm clumps (*SI Appendix*, Fig. S8*D*) (in small, medium, and large sizes), and incompletely dissociated sperm bundles (*SI Appendix*, Fig. S8*E*). Notably, the quantities of free eupyrene sperm and incompletely dissociated sperm bundles were significantly lower in two mutant strains than the WT strain (Fig. 4 *C* and *D*), indicating reduced storage of sperm in the spermatheca in the absence of PBP4 or Orco. Conversely, the quantities of free eupyrene and apyrene sperm and incompletely dissociated sperm bundles left in the spermatophore were significantly higher in the two mutant strains than in the WT strain (Fig. 4 *E* and *F*). These findings suggest that the impaired transfer of sperm from the spermatophore to spermatheca is a key mechanism contributing to the reduced sperm competition observed in *PBP4* and *Orco* knockout insects.

To assess the impact of *PBP4* and *Orco* knockouts on sperm motility, the motility of sperm from WT, *PBP4^−/−^*, and *Orco^−/−^* males was examined and documented in vitro. In Lepidoptera, eupyrene sperm exhibit very weak motility, whereas apyrene sperm exhibit vigorous motility (32, 33). Motile apyrene sperm were classified into two grades, grade I and grade II, based on the portion of flagellum length that oscillated. Grade I motile sperm were characterized by less than half of the flagellar length displaying oscillation, while grade II motile sperm oscillated with more than half of the flagellum (Movie S1). The total motile sperm ratios for WT, *PBP4^−/−^*, and *Orco^−/−^* males were 45.4, 40.9, and 40.7%, respectively (Fig. 4*G*), with no significant differences among the three groups. However, the ratios of grade II motile sperm from both *PBP4^−/−^* and *Orco^−/−^* males were significantly lower than in WT males (Fig. 4*G*), suggesting that the reduced sperm storage in the spermatheca was resulted from the decreased sperm motility.

### The OR Chemical Signaling System Mediates Sperm Activation in the Spermatophore.

**Fig. 5.**
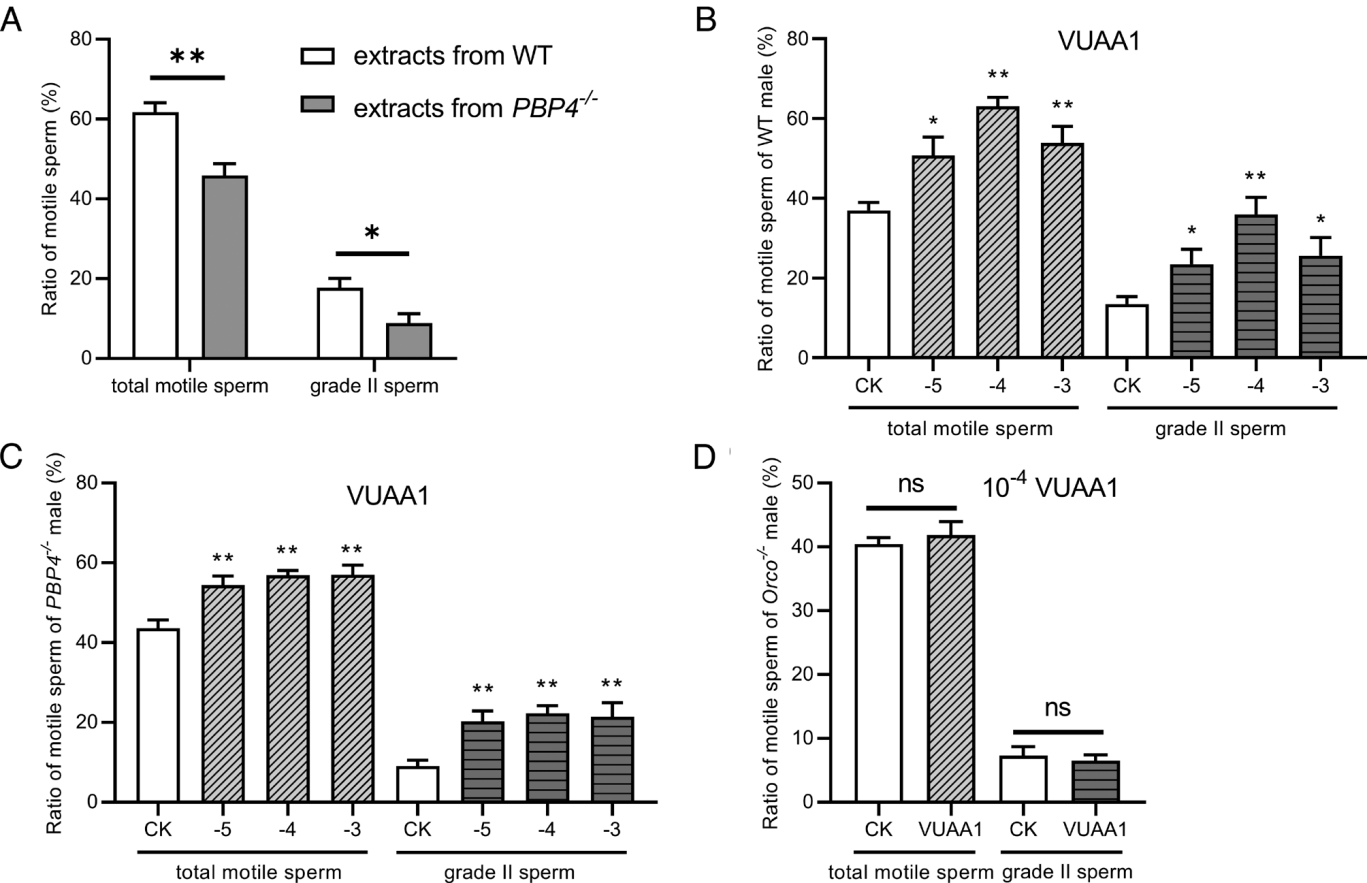
Crude extract of WT spermatophores and VUAA1 enhance the sperm motility. (*A*) The effect of crude extracts from WT and *PBP4^−/−^* male spermatophores on motility of sperms of *PBP4^−/−^* males. (*B*) The effect of VUAA1 on sperm motility of WT males. (*C*) The effect of VUAA1 on sperm motility of *PBP4^−/−^* males. (*D*) The effect of VUAA1 (10^−4^ M) on sperm motility of *Orco^−/−^* males. n = 10 (*A*–*D*). * indicates significant difference with the control (CK) (Student’s *t* test, *P < 0.05, **P < 0.01) (*A*–*C*); “ns” indicates no significant difference with the CK (Student’s *t* test, *P* < 0.05) (*D*).

The ability to bind low molecular weight substances, including signaling chemicals, nutrients, hormones, and pesticide molecules (34) endows OBPs with diverse roles. Based on the above results, we hypothesized that PBP4 binds and transports specific low molecular weight substances from the MAGs to the spermatophore, where the ligands can be released to activate the OR/Orco complex of the sperm, thereby triggering motility. To validate it, we first obtained crude extracts from WT and *PBP4^−/−^* male spermatophores and assessed their effects on sperm motility by In vitro sperm motility assay. As a result, the crude extracts from WT males significantly enhanced sperm motility in comparison with those from *PBP4^−/−^* males (Fig. 5*A*), suggesting the involvement of endogenous ligands of PBP4, released from PBP4 at this stage after mating, in enhancing sperm motility.

Further In vitro sperm motility assays also revealed that the Orco agonist VUAA1, at concentrations ranging from 10^−5^ to 10^−3^ M, significantly enhanced overall sperm motility and grade II motile sperm in WT males (Movie S2, 3), with the most pronounced activation at a concentration of 10^−4^ M (Fig. 5*B*). Further tests for sperm from *PBP4^−/−^* males, VUAA1 showed a similar enhancing effect on sperm motility (Fig. 5*C* and Movies S4 and S5). However, for sperm from *Orco^−/−^* males, 10^−4^ M of VUAA1 had no significant impact on the sperm motility (Fig. 5*D* and Movies S6 and S7), demonstrating that Orco ion channels (probably as Orco/ORx complexes) mediate activation of sperms.

### The Function of PBP4 Is Conserved in *Spodoptera* Species.

**Fig. 6.**
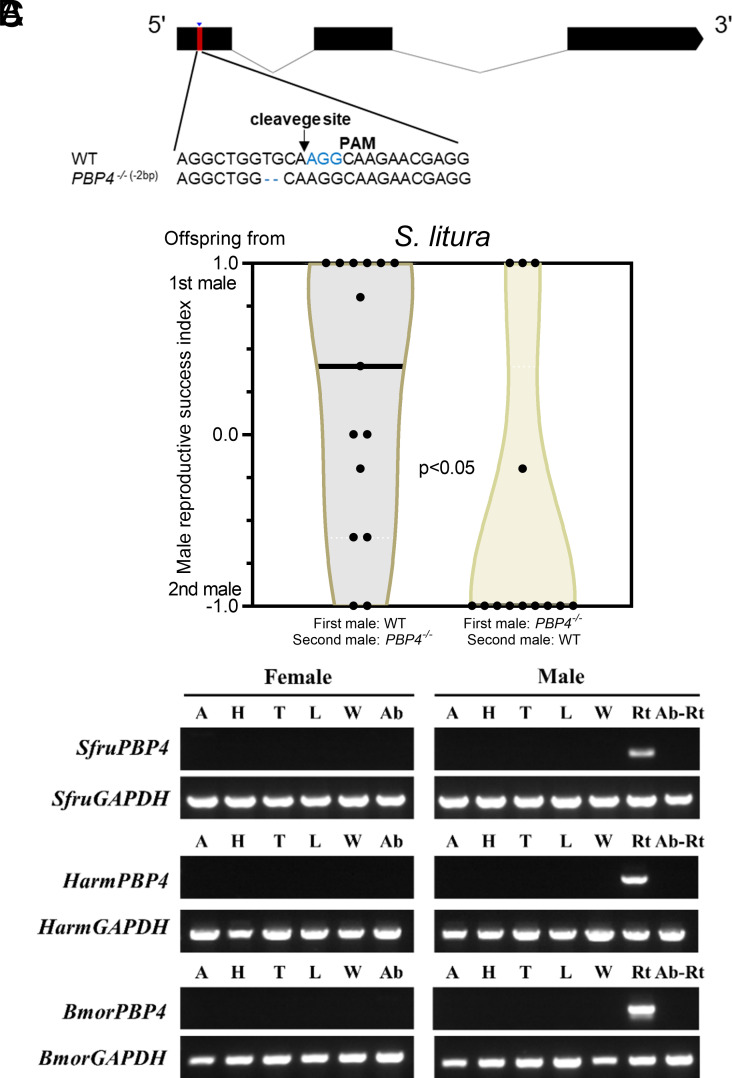
The function of PBP4 is conserved across *Spodoptera* species. (*A*) Schematic diagram of single-guide RNA (sgRNA) targeting site of *SlitPBP4*. The PAM sequence is in blue. The change in length is shown *Left* of each sequence (–, deletion). (*B*) Violin plots represent male reproductive success index. The difference of male success reproductive index after changing the order of mating was tested via the Mann–Whitney *U* test. (*C*) Semiquantitative analysis of PBP4 genes of *Spodoptera frugiperda*, *Helicoverpa armigera,* and *Bombyx mori*.

Phylogenetic analysis showed that *PBP4* genes from noctuid species form a distinct clade, alongside with *PBP-B* genes from three non-noctuid species (*BmorPBP4*, *MsexPBP4,* and *DplePBP1*) (*SI Appendix*, Fig. S1). To investigate whether the function of PBP4 is conserved across species, we conducted the same experiments in *S. litura*, a congeneric species of *S. exigua,* but exhibiting an opposite sperm precedence (the second male precedence) (30). Using CRISPR-Cas9 genome editing, we generated a *SlitPBP4* knockout strain (Fig. 6*A*). Double-mating experiments showed that *PBP4*-depleted males in *S. litura* exhibited reduced sperm competition compared to WT males (Fig. 6*B*), indicating that the function of PBP4 is conserved within the *Spodoptera* genus.

Despite their shared origin, noctuid *PBP4* genes differ significantly both in sequence length and identity from *PBP-B* genes in non-noctuid moths (*SI Appendix*, Fig. S10). We therefore in addition to the two *Spodoptera* species (21, 22) expanded the examination of tissue expression to two more noctuid representatives (*Helicoverpa armigera* and *S. frugiperda*) and one non-noctuid species, *B. mori,* We found that *PBP4* was specifically expressed in the reproductive tracts of males across all these species (Fig. 6*C*). It has been reported that *PBP4* in *M. sexta* shows up in the antennal transcriptome of both male and female adults, but that it appears to be highly expressed in Malpighian tubule of adult moths (8). However, experimental evidence of its function remains limited. Based on the data available, we hypothesize that the function of *PBP4* is conserved across noctuid species, while the *PBP-B* genes in some non-noctuid moths may have a similar function.

## Discussion

Proteins that are produced by the MAGs and transferred alongside semen to females during copulation play important roles in the physiology and behavior of mated female insects (35, 36). Here, we reveal that a PBP paralog (PBP4), specifically and highly expressed in MAGs, is essential for sperm competition via the OR pathway in moth species. This finding expands our understanding of the evolution and functional differentiation of PBPs as well as of MAG proteins, and also provides a target for the sterile insect technique used in pest control.

*S. exigua* PBP4 shares high sequence identity with PBPs expressed in the antenna, but is specifically expressed in MAGs and transferred to females in the male spermatophore during mating. MAG proteins of insects play various roles in female physiology, including forming a mating plug to prevent repeated mating (37), changing uterine conformation (38), and aiding in sperm storage and release (39). Notably, sex peptides (SPs) not only interact with sperm but also partially enter the female’s hemolymph circulation system, where they inhibit sex peptide sensory neurons that express the SP receptor (40, 41). SP is implicated to be involved in nearly all postmating responses in *Drosophila melanogaster* (42, 43). The injection of synthetic *D. melanogaster* SP into virgin females of the moth *H. armigera* has been shown to suppress pheromone production (44). The seminal fluid protein OBP10 in *H. armigera* is transferred to the female during mating and subsequently detected in the eggs, suggesting its potential role as a carrier for oviposition deterrents (45). However, we conducted a series of bioassays regarding mating rate, remating rate, egg laying, and egg hatchability and found that PBP4 had no effect on male and female physiology. Furthermore, transcriptome analysis and qPCR validation revealed that knocking out PBP4 had no effect on gene expression in the head and abdomen of females at various time intervals after mating. In addition, we found that PBP4 undergoes an enzymolysis upon its transfer to the female, and the enzyme for the enzymolysis is also a seminal fluid protein, resembling the phenomenon observed in the seminal fluid of *D. melanogaster* (46). These results indicate that the function of PBP4 is male-dependent, although it takes place in the female reproductive system.

For moths as well as other insects, it is common that females mate more than once, leading to sperm competition between males. Our results determined that contrary to most other moths (30, 31, 47), where the last male fertilizes most of the eggs, but similar to *Tetranychus urticae* (48) and *Pseudoplusia includens* (49), *S. exigua* exhibits a first male sperm precedence. Numerous factors influence sperm competition, such as sperm storage, sperm retention, the formation of mating plugs, postmating female receptivity, and sperm motility (50). Our study showed that *PBP4* in *S. exigua* plays an important role in sperm competition, as offspring sired by *PBP4* knockout males significantly decreased, and recovered when the PBP4 was rescued. Our study demonstrates that a PBP paralog is involved in reproductive physiology and no longer binds sex pheromones, completely different in comparison to PBP1-3. The PBP1-3 in *S. exigua* and other noctuid moths play important roles in the detection of female sex pheromones (51–53). Although phylogenetically closely related with other PBPs, PBP4 is obviously no longer a suitable name from the functional perspective.

It is well known that *Orco* is involved in the detection of odors as a coreceptor in antennae (54), however, the expression of *Orco* was also reported for nonolfactory tissues (55, 56). In particular, a study in *Anopheles gambiae* showed that Orco is expressed in sperm and comediates sperm motor activation with ORs (26). Furthermore in mammals, sperm ORs have been demonstrated to affect sperm motility (25, 57, 58). In our study, transcript and qPCR analyses revealed a significant upregulation of *Orco* in the reproductive tract of *PBP4^−/−^* males, and further assays using the *Orco^−/−^* strain confirmed that Orco is also involved in sperm competition. Therefore, we suppose that the function of PBP4 in sperm competition is achieved via Orco (likely ORx/Orco) by mediation of sperm motility, which is confirmed by our further investigation of sperm motility and sperm number in spermatophore and spermatheca.

The decline in sperm competition is generally attributed to a reduction in the number of sperm stored in the spermatheca (37, 59). Unlike *D. melanogaster* and *A. gambiae*, which generate only monomorphic sperm (26, 60), Lepidoptera females produce two morphologically distinct types of sperm, eupyrene, and apyrene sperm (28). The eupyrene sperm are nearly nonmotile, whereas apyrene sperm are motile and their motility facilitates the movement of eupyrene sperm (32, 33). Our present study demonstrates that apyrene sperm from *PBP4^−/−^* and *Orco^−/−^* males exhibit significantly lower motility than those from the wild-type males, as indicated by the ratio of grade II motile sperm. Consistently, the number of sperm was significantly reduced in the spermatheca, but higher numbers remained in the spermatophore females that mated with *PBP4^−/−^* and *Orco^−/−^* males compared to those mated with WT males.

Although PBP4 differs significantly in tissue specificity and function from PBP1-3, these PBPs share a similar mode of action, PBP-OR, and more likely PBP-ligand-ORx/Orco. In our sperm motility assay, crude extracts from WT male spermatophores indeed significantly enhanced sperm motility than extracts from *PBP4^−/−^* male spermatophores. This suggests that ligands of PBP4 contribute to the sperm activation, as PBP4 itself has already been degraded at the time. It is likely that PBP4 carries the endogenous ligand from male to female in the spermatophore, where PBP4 releases ligand after its C-terminus (about 30 amino acids) is cleaved. With the removal of the C-terminus, the theoretical isoelectric point (pI) of PBP4 decreases from 8.87 to 5.66, which induces conformational change of the protein and further release of the ligand. Unfortunately, we failed to identify the ligand in the present study.

VUAA1 has been determined as an agonist of the Orco across various insect species (23, 24), functioning through inhibition of Kv2 potassium channels (23). The current induced by VUAA1 is dependent on Orco alone, rather than the Orco/ORx complex, and is similar in shape to that evoked by odorants (24). Here, VUAA1 enhanced the motility of sperm from WT and *PBP4^−/−^* males but had no effect on sperm from *Orco^−/−^* males, strongly indicating that Orco ion channel mediated sperm activation. Unlike what has been found in vertebrates that use a single OR for olfaction (61), insects use the heterotetramer ORx/Orco as a functional unit (62, 63), and it has been shown that Orco and ORx comediates sperm motor activation in *A. gambiae* (24). Furthermore, 10 ORs are found to be expressed in the male reproductive tract of *S. exigua* (64). However, we cannot exclude that Orco alone functions as the ion channel of sperm. Future studies deserve to identify the possible ligand of PBP4, and to reveal whether and which specific OR(s) might be involved. The conserved mode of action in olfaction of sex pheromones and sperm motility reflects the parsimony in biological pathways, providing an insight into gene evolution and functional differentiation. Based on our phylogenetic analysis and functional validation, we hypothesize that the function of *PBP4* is conserved across noctuid moths. Further studies are needed to test our hypothesis that the PBP-B genes in non-noctuid moths fulfill the same role.

The observed upregulation of *Orco* expression in *PBP4^−/−^* males could be attributed to genetic compensation mechanisms triggered by mutated mRNA, a phenomenon commonly occurring among homologous genes (65). This genetic robustness may arise from intricately regulated cellular networks, including metabolic, signaling, and transcriptional pathways (66). One example is that the knocking out of *OR56* in silkworms leads to up- and downregulation of multiple olfactory genes including ORs, OBPs, and CSPs (67). Overall, our study uncovers a role for PBP4 in sperm motility and competition in *S. exigua*, suggesting the conservation of this function across noctuid moths and providing insights into the molecular mechanisms governing sperm motility and sperm competition in Lepidoptera.

## Materials and Methods

### Insect Rearing.

All moths were reared at 26 °C, 65 to 75% relative humidity, and 14:10 h light/dark photoperiod in an insectary. The larvae were fed with artificial diet, pupae were sexed and transferred to different cages for eclosion, and adults were fed with 10% honey solution.

### Tissue Collection.

To assess the expression pattern of *SexiPBP4*, various tissues were isolated from 2-d-old (2d) virgin male and female moths, with each replicate consisting of 80 antennae, 20 heads, 20 thoraxes, 30 wings, 20 legs, 10 reproductive tracts, and 10 remaining abdomens. To further investigate protein expression, the testis, seminal duct, seminal vesicle, MAGs, and ejaculatory duct were dissected from five 2d virgin males. Furthermore, 40 antennae were collected from both virgin and 6 h postmated (6 hpm) males and females for western blot analysis.

To investigate distribution of PBP4 protein, five abdomens and five reproductive tracts were collected from virgin males and females, respectively, and to determine the degrading dynamics of the PBP, five spermatophores were collected within a time frame of 0 to 8 h after mating. To determine the origin of the enzyme responsible for cleaving the PBP4 protein within spermatophores, spermatophores were collected by two methods. The first method was to collect spermatophores from the abdomen of females at 0 h after mating completion, in which the spermatophores were produced and stored in the female’s abdomen; and the second was to allow the moths to mate for 5 min, and then, the female was removed while the male continued producing a spermatophore that was collected after its completion. In the second method, the spermatophores were produced by males that were detached with females and thus contained nothing from females. Based on whether the spermatophores were produced in the female’s abdomen or not, the first and second methods for spermatophore collecting were designated as in vivo and in vitro methods, respectively.

To assess the expression pattern of *SfruPBP4*, *HarmPBP4,* and *BmorPBP4*, 20 antennae, 5 heads, 5 thoraxes, 10 wings, 10 legs, 5 reproductive tracts, and 5 remaining abdomens were isolated from 2d virgin male and female moths.

### RNA Isolation, cDNA Synthesis, and Quantitative Real-Time RT-PCR (qRCR).

Total RNA was isolated from tissues utilizing Trizol Reagent (Invitrogen) following the manufacturer’s protocol. Subsequently, the first single-strand cDNAs were synthesized using PrimeScript™ RT reagent Kit with gDNA Eraser (TaKaRa, China).

The qRCR was performed using the TB Green® Premix Ex Taq™ (TaKaRa, China) on a QuantStudio 6 Flex Real-Time PCR System (Applied Biosystems). The cycling conditions for qPCR consisted of an initial denaturation step at 95 °C for 30 s, followed by 40 cycles of denaturation at 95 °C for 5 s and annealing/extension at 60 °C for 34 s. The housekeeping gene glyceraldehyde-3-phosphate dehydrogenase (GAPDH) was utilized as an internal control. Three independent biological replicates were measured for each gene, and the gene expression levels were analyzed by 2^-△Ct^ method. The primers used are shown in *SI Appendix*, Table S1.

### Western Blot Analysis.

Tissues were homogenized using 200 µL of RIPA lysis buffer in a 2 mL Eppendorf tube, followed by centrifugation at 13,000 g for 10 min after incubation on ice for 30 min. Subsequently, 20 µL of 5× loading buffer was added to 80 µL of the supernatant, and the mixture was boiled for 10 min.

The samples were subjected to 15% denaturing polyacrylamide gel electrophoresis (PAGE) and transferred to a PVDF membrane. The membrane was then blocked with 5% nonfat milk for 1 h at room temperature and incubated with primary antibody solution overnight at 4 °C, and subsequently with secondary antibody solution for 1 h at room temperature. The primary antibodies used were rabbit anti-PBP4 (1:1,000 dilution, GenScript, China) and β-Actin Rabbit mAb (1:4000 dilution, ABclonal, China), while the secondary antibody was HRP Goat Anti-Rabbit IgG (1:5000 dilution, ABclonal, China).

### Prokaryotic Expression of SexiPBP4 Protein in *Escherichia* coli.

Following the evaluation of various expression vectors, it was determined that the MBP fusion protein was able to maintain a relatively stable soluble state. The SexiPBP4 coding sequence without signal peptide was subcloned into pET28a-MBP vector downstream of the mbp fragment to construct the recombinant plasmids pET28a-MBP-PBP4. The plasmid was transformed into *E. coli* BL21 (DE3) and induced to express fusion proteins MBP/PBP4 with 0.1 mM isopropyl‐β‐D‐thiogalactoside for 24 h at 16 °C. The expressed protein was purified by Ni-NTA agarose magnetic beads and the fusion tag was cleaved from the protein by enterokinase. The protein was detected by 15% SDS-PAGE.

### Fluorescence Competition Binding Assay.

To determine the binding affinities of SexiPBP4 to four sex pheromones (*SI Appendix*, Table S2). The protein (in a pH 7.4 buffer containing 50 mM Tris, 150 mM NaCl) was titrated with aliquots of 500 μM N-phenyl1-naphthylamine (1-NPN) dissolved in methanol to final concentrations of 2 to 20 μM. The fluorescence was measured at 415 nm upon excitation at 337 nm. Having observed the binding of 1-NPN, bindings of metabolites to PBP4 protein were checked by monitoring the displacement of PBP4-bound 1-NPN by metabolites (2 to 20 μM). The dissociation constant (Ki) for each compound was determined from the IC_50_ values using the following formula: Ki = IC_50_/[1 + (1-NPN)/K_1-NPN_], where (1-NPN) is the free concentration of 1-NPN, and K_1-NPN_ is the dissociation constant for the protein/1-NPN complex.

### CRISPR-Cas9-Based Genome Editing.

The sgRNAs were designed to target the first coding exon of *SexiPBP4* and *SlitPBP4*, and the second coding exon of *SexiOrco* using CRISPR Design (http://crispor.tefor.net/), and synthesized with the Precision gRNA Synthesis Kit (Thermo Fisher Scientific). The primers used are shown in *SI Appendix*, Table S1.

Equal numbers of 2d adult males and females were paired together for one day, after which the females were placed individually in a plastic container. Eggs were then collected every 30 min and affixed to glass slides. Each egg was injected with a mixture of 200 ng/μL sgRNA and 300 ng/μL Cas9 protein (Thermo Fisher Scientific) within 2 h after oviposition using an Eppendorf FemtoJet and InjectMan NI 2 microinjection system (Eppendorf, Germany). The injected eggs were incubated at 25 °C with 90% humidity for 24 h before being returned to standard conditions for hatching.

Adult G0 individuals were crossed with wild-type moths to produce G1 offspring. The G1 larvae were prescreened through direct sequencing following the extraction of genomic DNA using DNAiso Reagent (TaKaRa, China). G1 adults were then crossed with wild-type moths again. After laying eggs, the genotypes of each G1 moth were determined through sequencing. Those with mutated genotypes resulting in loss-of-function proteins were raised as G2 larvae. Homozygous G2 adults were obtained by sequencing the final larval exuviae (68) and subsequently crossed with G2 adults of the same genotype to establish a homozygous mutated strain.

### pBac-Mediated Transposition.

To investigate the utilization pattern of sperm from two males that mated with a same female in *S. exigua*, a transgenic insect strain expressing red fluorescent protein was generated. The red fluorescent protein expression plasmid vector pBac[3xp3-EGFP; hr5IE1-DsRed2] designated “pBac[DsRed2]” was constructed. The 3xP3 fragment was synthesized by GenScript (Nanjing, China). EGFP, hrIE1, and DsRed2 fragments were cloned from *piggyBac* vector donated from Dr. Wang (*CAS Center for Excellence in molecular Plant Sciences, Shanghai, China*). The three amplified fragments were inserted into the *piggyBac* vector through homologous recombination to create the pBac[DsRed2] vector. The primers utilized in this process are detailed in *SI Appendix*, Table S1.

To rescue the expression of *SexiPBP4* in *PBP4*-knockout strain, a transgenetic strain was generated by *piggyBac* system. The rescue plasmid vector pBac[3xp3-EGFP; PBP4 promotor-PBP4:his6] designated as “pBac[PBP4:his6]”, was constructed. Briefly, the 1274 bp upstream DNA sequence of the 5′-UTR of the *PBP4* gene was selected as the promoter sequence and amplified from genome; PBP4:his6 fragment was cloned from cDNA with a primer pair including his6 sequence; the two amplified fragments were inserted to *piggyBac* vector by homologous recombination to generate the pBac[PBP4:his6] vector. The primers utilized in this process are detailed in *SI Appendix*, Table S1.

The complete coding sequence of the Hyperactive *piggyBac* transposase (hyPBase) gene, including the T7 promoter, was synthesized by GenScript (Nanjing, China) and subsequently inserted into the pUC19 vector. The hyPBase mRNA was transcribed using the mMESSAGE mMACHINE® T7 Kit (Thermo Fisher Scientific). Eggs were collected promptly within 2 h after oviposition for microinjection. A mixture of 300 ng/μL of *piggyBac* vector and 350 ng/μL of hyPBase mRNA was microinjected into individual eggs using an Eppendorf FemtoJet and InjectMan NI 2 microinjection system.

Adult G0 individuals were randomly paired in small groups for mating, and one day later, females were moved individually to plastic cages for oviposition. The G1 larvae exhibiting the 3xP3-EGFP (green fluorescence in the eyes and optic nerves) or hr5IE1-DsRed2 (red fluorescence in the whole body) phenotypes were screened at 4 to 5 d after hatching using a fluorescence stereomicroscope (NikonSMZ25, Japan). Positive individuals were selected, raised, and subsequently outcrossed to wild-type moths. The integration site of the transgene insertion in the positive individuals was identified using high-efficiency TAIL-PCR as described in a previous study (69). The larvae containing a single copy of the *piggyBac*-mediated transgene insertion were reared to screen homozygous. The chromosomal locations of the transgene insertions were detailed in *SI Appendix*, Table S4. The reproductive fitness phenotype of the transgenetic strains is detailed in *SI Appendix*, Table S5.

### Behavioral Assays.

#### Mating and remating.

Mating trials were conducted in mesh cages (28 cm in diameter, 32 cm in height), with one cage containing 15 pairs (15 males and 15 females) of 1d virgin moths. After 24 h, 120 females were dissected to count the number of females containing spermatophore, while the remaining 120 females were transferred to a new cage to engage in remating for an additional day with fresh 1d males. Subsequent dissections were performed to tally the number of females containing two spermatophores.

Females of *S. exigua* normally mate only once in a day, and each mating results in the transfer of one spermatophore. Therefore, mating times can be determined according to the number of spermatophores in the females.

#### Egg laying and hatchability.

The 1d moths were paired in a mesh cage for one day; then, females were transferred individually to new cages to lay eggs for four days, which encompasses almost the entire egg-laying period. The numbers of eggs and hatched larvae were documented.

#### Calling rhythm.

Female moths that successfully mated in the mesh cage were moved individually to 250 mL hyaline plastic cups. Calling behavior (ovipositor extension) was observed in the dark period of the following day at half-hour intervals.

#### Pheromone content measurement.

After 2 to 3 h of copulation, pheromone glands were extracted from 2 d females. Briefly, five pheromone gland–ovipositor complexes excised by microscissors were immersed with 30 μL hexane (containing 0.5 ng/μL 11:Ac as internal standard) for 30 min to obtain the pheromone extracts. The extract samples were analyzed by GC (Bruker 456GC, Germany). The injector temperature was set at 220 °C, and the detector temperature was at 250 °C; the oven temperature initially set at 60 °C for 2 min, then raised at a rate of 5 °C/min to 250 °C, and maintained for 5 min.

#### Sperm competition assay.

The sperm utilization pattern of the female was determined by checking genotype of the offspring larvae that was obtained from females sequentially mated with gene-edited male moths and wild-type moths. Four sets of experiment were carried out (W = wild type, G = gene-edited): Set I (W female × W male × G male), Set II (W female × G male × W male), Set III (G female × W male × G male), Set IV (G female × G male × W male). The 1 d moths were paired for the first mating, and 24 h later (for *S. exigua*) or 48 h later (for *S. litura*, the remating success after an interval of only 24 h was poor) the second mating was conducted. Eggs were collected every 24 h for two consecutive days and were reared to larvae. The paternity for each larvae was determined through sequencing (16 larvae per repeat, 8 to 16 repeats for each treatment) or fluorescence stereomicroscopy (22 to 95 larvae per repeat, 19 to 27 repeats for each treatment). The male reproductive success index (offspring of 1st male - offspring of 2nd male/ all offspring) was used to evaluate the efficacy of sperm competition.

### RNA-Seq and Data Analysis.

To explore differences in gene expression, reproductive tracts (10) were collected from 1 d WT and *PBP4*^−/−^ males, while heads (20) and abdomens (10) were excised from females at 2, 6, and 24 h after mating with 1d WT or *PBP4*^−/−^ males. Total RNA was extracted from the tissues using Trizol Reagent (Invitrogen), and transcriptomic analysis was conducted at Tianjin Novogene Bioinformatics Technology (Tianjin, China). The final cDNA library was produced after purification with AmPure XP beads and sequenced on Illumina HiSeq platform. Differential gene expression was carried out using DEseq with cutoffs of log2 fold change >2 and adjusted P-values <0.05 within replicates applied.

### Sperm Counting.

Virgin male and female moths were placed in a mesh cage for mating 1 d after emergence. After mating, females were snap frozen in liquid nitrogen at 0 and 16 h after the mating. Spermathecae and spermatophores were dissected and placed in 1 mL insect saline (0.75% NaCl, 0.05% KCl, 0.015% CaCl_2_, 0.005% NaHCO_3_, 0.004% NaH_2_PO_4_, and 0.02% glucose). In accordance with a previously described method (70), the sperm storage organs were ruptured and homogenized, and then, eupyrene and apyrene sperm were counted in a hemacytometer. Eupyrene sperm were characterized as being long, thick, and straight, while apyrene sperm were short, thin, and curved.

### Electroantennogram (EAG) Recording.

The EAG responses of WT and *Orco^−/−^* male antennae to six common plant volatiles and two main sex pheromone components (*SI Appendix*, Table S7) were measured with hexane (the solvent) as the control. Briefly, the antenna of a virgin male adult (2d) was cut off and connected to the two recording electrodes by gel (SPECTRA 360). The 10 μL of each odorant solution (1.0 μg/μL for plant odors, and 0.1 μg/μL for pheromones) was loaded onto a filter paper (2.5 cm × 0.8 cm). After volatilizing for 3 min, the paper strip was inserted into a Pasteur pipette. The amplitude of the EAG response was recorded and digitized with IDAC-1 controller (Syntech, Germany). EAG amplitudes were calculated by subtracting the solvent response.

### Sperm Motility Analysis.

Sperm flagellum activation in response to the Orco-specific agonist, 2-((4-ethyl-5-(pyridin-3-yl)-4H-1,2,4-triazol-3-yl)thio)-N-(4-ethylphenyl) acetamide (VUAA1) (Sigma-Aldrich, Germany), and the crude extracts were measured using sperm dissected from the spermatophores of 2–3d females 2 h after the end of mating. Crude extracts were also collected from spermatophores of 2–3d females 2 h after the end of mating. Assay buffer [145 mM NaCl, 4 mM KCl, 1 mM MgCl_2_, 1.3 mM CaCl_2_, 5 mM D-glucose, 10 mM 4-(2-Hydroxyethyl)piperazine-1-ethanesulfonic acid (HEPES), pH 7.4] was added to the semen at a ratio of 15 µL/spermatophore. The crude extracts were centrifuged at 12,000 g for 20 min to remove sperm. Subsequently, the supernatant was incubated at 90 °C for 10 min to denature proteins. Sperm collected from a spermatophore were released into 15 µL of assay buffer or crude extracts and gently mixed by pipetting. 4.5 µL solution was mixed with 0.5 µL of test chemicals dissolved in DMSO or assay buffer and then transferred to a clean glass microscope slide. A coverslip was placed on top of the microscope slide and then observed under an Axio observer 3 microscope (Carl Zeiss, Germany). The sperm motility of scattered sperm was observed from 3 different regions of the slide, with each compound being repeated for 10 times (slides). The proportion of motile sperm and different motility grades (Movie S1) were recorded. Grade I motile sperm was designated as more than half of the flagellum oscillating, and grade II motile sperm was less than half of the flagella oscillating.

### Construction of the PBP/GOBP Phylogenetic Tree.

The PBP and GOBP amino acid sequences were collected from 4 Noctuoidae species (*S. exigua*, *S. litura*, *S. frugiperda*, and *H. armigera*), one Carposinidae species (*Carposina sasakii*), 1 Tortricidae species (*Grapholita funebrana*), 1 Geometridae species (*Ectropis obliqua*), 3 Pyralidae species (*Ostrinia nubilalis*, *Ostrinia furnacalis*, and *Chilo suppressalis*), one Papilionidae species (*Danaus plexippus*), one Plutellidae species (*Plutella xylostella*), and 4 Bombycoidea species (*Antheraea Polyphemus*, *Antheraea pernyi*, *Manduca sexta*, and *B. mori*). The signal peptides predicted using SignalP-5.0 (https://services.healthtech.dtu.dk/services/SignalP-5.0/) were removed, and then aligned using ClustalW with default parameters. The maximum-likelihood tree was constructed by MEGA-X, and Bootstrap support values were calculated with 1,000 replicates. The phylogenetic tree was modified by iTOL (https://itol.embl.de/).

### Statistical Analysis.

Comparisons between two groups were analyzed using an unpaired two-tailed Student’s *t* test or Mann–Whitney *U* test. For comparisons of more than two groups, Welch and Brown–Forsythe one-way ANOVA with a relevant post hoc test was performed. All statistical analyses were conducted using SPSS 27.0 software, and a *P* value < 0.05 was considered statistically significant.

## Supplementary Material

Appendix 01 (PDF)

Movie S1. The depiction of different motility grades for apyrene sperm.

Movie S2. The apyrene sperm motility in WT males.

Movie S3. The apyrene sperm motility in WT males after addition of 10^-4^ M VUAA1.

Movie S4. The apyrene sperm motility in *PBP4^-/-^* males.

Movie S5. The apyrene sperm motility in *PBP4^-/-^* males after addition of 10^-4^ M VUAA1.

Movie S6. The apyrene sperm motility in *Orco^-/-^* males.

Movie S7. The apyrene sperm motility in *Orco^-/-^* males after addition of 10^-4^ M VUAA1.

## Data Availability

The RNA-seq raw data are available from NCBI with BioProject ID: PRJNA1234362 (https://www.ncbi.nlm.nih.gov/bioproject/PRJNA1234362) (71). All other data are included in the article and/or supporting information.

## References

[1] B. S. Hansson, M. C. Stensmyr, Evolution of insect olfaction. Neuron **72**, 698–711 (2011). 22153368 10.1016/j.neuron.2011.11.003

[2] A. T. Groot, T. Dekker, D. G. Heckel, The genetic basis of pheromone evolution in moths. Annu. Rev. Entomol. **61**, 99–117 (2016). 26565898 10.1146/annurev-ento-010715-023638

[3] C. Wang, Y.-H. Li, L. Wang, B. Yang, G.-R. Wang, Development of a new sex attractant via the peripheral coding of pheromones in Mythimna loreyi. J. Agric. Food Chem. **71**, 2795–2803 (2023). 36726240 10.1021/acs.jafc.2c07131

[4] R. G. Vogt, L. M. Riddiford, Pheromone binding and inactivation by moth antennae. Nature **293**, 161–163 (1981). 18074618 10.1038/293161a0

[5] J. E. Allen, K. W. Wanner, Asian corn borer pheromone binding protein 3, a candidate for evolving specificity to the 12-tetradecenyl acetate sex pheromone. Insect Biochem. Mol. Biol. **41**, 141–149 (2011). 21056664 10.1016/j.ibmb.2010.10.005

[6] D. Cao , Identification of candidate olfactory genes in Chilo suppressalis by antennal transcriptome analysis. Int. J. Biol. Sci. **10**, 846 (2014). 25076861 10.7150/ijbs.9297 PMC4115196

[7] D.-P. Gong, H.-J. Zhang, P. Zhao, Q.-Y. Xia, Z.-H. Xiang, The odorant binding protein gene family from the genome of silkworm, *Bombyx mori*. BMC Genomics **10**, 1–14 (2009). 19624863 10.1186/1471-2164-10-332 PMC2722677

[8] R. G. Vogt, E. Große-Wilde, J.-J. Zhou, The lepidoptera odorant binding protein gene family: Gene gain and loss within the GOBP/PBP complex of moths and butterflies. Insect Biochem. Mol. Biol. **62**, 142–153 (2015). 25784631 10.1016/j.ibmb.2015.03.003

[9] Y.-Q. Song, J.-F. Dong, H.-L. Qiao, J.-X. Wu, Molecular characterization, expression patterns and binding properties of two pheromone-binding proteins from the oriental fruit moth, Grapholita molesta (Busck). J. Integr. Agric. **13**, 2709–2720 (2014).

[10] D. Jing , Molecular characterization and volatile binding properties of pheromone binding proteins and general odorant binding proteins in Conogethes pinicolalis (Lepidoptera: Crambidae). Int. J. Biol Macromol. **146**, 263–272 (2020). 31923484 10.1016/j.ijbiomac.2019.12.248

[11] V. Rojas , Analysis of the grapevine moth Lobesia botrana antennal transcriptome and expression of odorant-binding and chemosensory proteins. Comp. Biochem. Physiol. Part. D. Genomics Proteomics. **27**, 1–12 (2018). 29727827 10.1016/j.cbd.2018.04.003

[12] H. G. Consortium, Butterfly genome reveals promiscuous exchange of mimicry adaptations among species. Nature **487**, 94–98 (2012). 22722851 10.1038/nature11041 PMC3398145

[13] Y.-N. Zhang , Molecular identification and expression patterns of odorant binding protein and chemosensory protein genes in *Athetis lepigone* (Lepidoptera: Noctuidae). PeerJ **5**, e3157 (2017). 28382236 10.7717/peerj.3157 PMC5376112

[14] W.-M. Xiu, S.-L. Dong, Molecular characterization of two pheromone binding proteins and quantitative analysis of their expression in the beet armyworm. *Spodoptera exigua* Hübner. J. Chem. Ecol. **33**, 947–961 (2007). 17393279 10.1007/s10886-007-9277-2

[15] S.-H. Gu, J.-J. Zhou, G.-R. Wang, Y.-J. Zhang, Y.-Y. Guo, Sex pheromone recognition and immunolocalization of three pheromone binding proteins in the black cutworm moth Agrotis ipsilon. Insect Biochem. Mol. Biol. **43**, 237–251 (2013). 23298680 10.1016/j.ibmb.2012.12.009

[16] T. Zhang, S. Gu, K. Wu, Y. Zhang, Y. Guo, Construction and analysis of cDNA libraries from the antennae of male and female cotton bollworms Helicoverpa armigera (Hübner) and expression analysis of putative odorant-binding protein genes. Biochem. Biophys. Res. Commun. **407**, 393–399 (2011). 21396914 10.1016/j.bbrc.2011.03.032

[17] W.-M. Xiu, Y.-Z. Zhou, S.-L. Dong, Molecular characterization and expression pattern of two pheromone-binding proteins from Spodoptera litura (Fabricius). J. Chem Ecol. **34**, 487–498 (2008). 18347871 10.1007/s10886-008-9452-0

[18] Y.-Y. Zhang , Molecular mechanism of sex pheromone perception in male Mythimna loreyi revealed by in vitro system. Pest Manag. Sci. **80**, 744–755 (2024). 37779104 10.1002/ps.7806

[19] J.-Y. Jin, Z.-Q. Li, Y.-N. Zhang, N.-Y. Liu, S.-L. Dong, Different roles suggested by sex-biased expression and pheromone binding affinity among three pheromone binding proteins in the pink rice borer, Sesamia inferens (Walker)(Lepidoptera: Noctuidae). J. Insect Physiol. **66**, 71–79 (2014). 24862154 10.1016/j.jinsphys.2014.05.013

[20] X.-T. Dong , CRISPR/Cas9-mediated PBP1 and PBP3 mutagenesis induced significant reduction in electrophysiological response to sex pheromones in male Chilo suppressalis. Insect Sci. **26**, 388–399 (2019). 29058383 10.1111/1744-7917.12544 PMC7379591

[21] J.-B. Sun, N.-Y. Liu, S.-M. Li, Q. Yan, S.-L. Dong, Molecular cloning, tissue expression profiling and binding characterization of the pheromone binding protein SlitPBP4 from Spodoptera litura (Lepidoptera: Noctuidae). Acta Ent. Sin. **61**, 657–667 (2018).

[22] S. Liu , Cloning and expression profile analysis of four pheromone-binding protein genes in the fall armyworm, *Spodoptera frugiperda*. J. Environ. Entomol. **42**, 583–592 (2020).

[23] L. Yang , Bioactivities and modes of action of VUAA1. Pest Manag. Sci. **77**, 3685–3692 (2021). 32741076 10.1002/ps.6023

[24] P. L. Jones, G. M. Pask, D. C. Rinker, L. J. Zwiebel, Functional agonism of insect odorant receptor ion channels. Proc. Natl. Acad. Sci. U.S.A. **108**, 8821–8825 (2011). 21555561 10.1073/pnas.1102425108 PMC3102409

[25] M. Spehr , Identification of a testicular odorant receptor mediating human sperm chemotaxis. Science **299**, 2054–2058 (2003). 12663925 10.1126/science.1080376

[26] R. J. Pitts, C. Liu, X. Zhou, J. C. Malpartida, L. J. Zwiebel, Odorant receptor-mediated sperm activation in disease vector mosquitoes. Proc. Natl. Acad. Sci. U.S.A. **111**, 2566–2571 (2014). 24550284 10.1073/pnas.1322923111 PMC3932880

[27] L. B. Vosshall, Olfaction: Attracting both sperm and the nose. Curr. Biol. **14**, R918–R920 (2004). 15530382 10.1016/j.cub.2004.10.013

[28] M. Friedländer, R. K. Seth, S. E. Reynolds, Eupyrene and apyrene sperm: Dichotomous spermatogenesis in Lepidoptera. Adv. In. Insect Phys. **32**, 206–308 (2005).

[29] L. Qian , SPSL1 is essential for spermatophore formation and sperm activation in *Spodoptera frugiperda*. PLoS Genet. **19**, e1011073 (2023). 38048348 10.1371/journal.pgen.1011073 PMC10721193

[30] R. K. Seth, J. J. Kaur, D. K. Rao, S. E. Reynolds, Sperm transfer during mating, movement of sperm in the female reproductive tract, and sperm precedence in the common cutworm Spodoptera litura. Physiol. Entomol. **27**, 1–14 (2002).

[31] S. Yan , Sperm storage and sperm competition in the Helicoverpa armigera (Lepidoptera: Noctuidae). J. Econ. Entomol. **106**, 708–715 (2013). 23786058 10.1603/ec12402

[32] S. Chen , Dysfunction of dimorphic sperm impairs male fertility in the silkworm. Cell. Discov. **6**, 60 (2020). 32963806 10.1038/s41421-020-00194-6 PMC7477584

[33] H. Sakai , Dimorphic sperm formation by Sex-lethal. Proc. Natl. Acad. Sci. U.S.A. **116**, 10412–10417 (2019). 31036645 10.1073/pnas.1820101116 PMC6535010

[34] P. Pelosi, I. Iovinella, J. Zhu, G. Wang, F. R. Dani, Beyond chemoreception: Diverse tasks of soluble olfactory proteins in insects. Biol. Rev. Camb. Philos. Soc. **93**, 184–200 (2018). 28480618 10.1111/brv.12339

[35] K. Ravi Ram, L. K. Sirot, M. F. Wolfner, Predicted seminal astacin-like protease is required for processing of reproductive proteins in Drosophila melanogaster. Proc. Natl. Acad. Sci. U.S.A. **103**, 18674–18679 (2006). 17116868 10.1073/pnas.0606228103 PMC1693721

[36] B. R. Hopkins, J. C. Perry, The evolution of sex peptide: Sexual conflict, cooperation, and coevolution. Biol. Rev. Camb. Philos. Soc. **97**, 1426–1448 (2022). 35249265 10.1111/brv.12849 PMC9256762

[37] N. C. Brown , The seminal odorant binding protein Obp56g is required for mating plug formation and male fertility in *Drosophila melanogaster*. eLife **12**, e86409 (2023). 38126735 10.7554/eLife.86409 PMC10834028

[38] F. W. Avila, M. F. Wolfner, Acp36DE is required for uterine conformational changes in mated Drosophila females. Proc. Natl. Acad. Sci. U.S.A. **106**, 15796–15800 (2009). 19805225 10.1073/pnas.0904029106 PMC2747198

[39] F. W. Avila, L. K. Sirot, B. A. LaFlamme, C. D. Rubinstein, M. F. Wolfner, Insect seminal fluid proteins: Identification and function. Annu. Rev. Entomol. **56**, 21–40 (2011). 20868282 10.1146/annurev-ento-120709-144823 PMC3925971

[40] K. R. Ram, M. F. Wolfner, A network of interactions among seminal proteins underlies the long-term postmating response in Drosophila. Proc. Natl. Acad. Sci. U.S.A. **106**, 15384–15389 (2009). 19706411 10.1073/pnas.0902923106 PMC2741260

[41] Y. J. Kim , MIPs are ancestral ligands for the sex peptide receptor. Proc. Natl. Acad. Sci. U.S.A. **107**, 6520–6525 (2010). 20308537 10.1073/pnas.0914764107 PMC2851983

[42] E. Kubli, Sex-peptides: Seminal peptides of the *Drosophila* male. Cell. Mol. Life. Sci. **60**, 1689–1704 (2003). 14504657 10.1007/s00018-003-3052 PMC11146071

[43] G.-X. Guan, X.-P. Yu, D.-T. Li, Post-mating responses in insects induced by seminal fluid proteins and octopamine. Biology **12**, 1283 (2023). 37886993 10.3390/biology12101283 PMC10604773

[44] Y. Fan, A. Rafaeli, C. Gileadi, E. Kubli, S. W. Applebaum, Drosophila melanogaster sex peptide stimulates juvenile hormone synthesis and depresses sex pheromone production in *Helicoverpa armigera*. J. Insect Physiol. **45**, 127–133 (1999). 12770380 10.1016/s0022-1910(98)00106-1

[45] Y.-L. Sun, L.-Q. Huang, P. Pelosi, C.-Z. Wang, Expression in antennae and reproductive organs suggests a dual role of an odorant-binding protein in two sibling *Helicoverpa* species. PLoS One **7**, e30040 (2012). 22291900 10.1371/journal.pone.0030040 PMC3264552

[46] B. A. LaFlamme, K. Ravi Ram, M. F. Wolfner, The *Drosophila* melanogaster seminal fluid protease “seminase” regulates proteolytic and post-mating reproductive processes. PLoS Genet. **8**, e1002435 (2012). 22253601 10.1371/journal.pgen.1002435 PMC3257295

[47] C. W. Lamunyon, Determinants of sperm precedence in a noctuid moth *Heliothis virescens*: A role for male age. Ecol. Entomol. **26**, 388–394 (2001).

[48] L. R. Rodrigues, A. R. Figueiredo, T. Van Leeuwen, I. Olivieri, S. Magalhães, Costs and benefits of multiple mating in a species with first-male sperm precedence. J. Anim. Ecol. **89**, 1045–1054 (2020). 31872443 10.1111/1365-2656.13171

[49] L. Mason, D. Pashley, Sperm competition in the soybean looper (Lepidoptera: Noctuidae). Ann. Entomol. Soc. Am. **84**, 268–271 (1991).

[50] A. Civetta, J. M. Ranz, Genetic factors influencing sperm competition. Front. Genet. **10**, 820 (2019). 31572439 10.3389/fgene.2019.00820 PMC6753916

[51] N.-Y. Liu, C.-C. Liu, S.-L. Dong, Functional differentiation of pheromone-binding proteins in the common cutworm Spodoptera litura. Comp. Biochem. Physiol. A. Mol. Integr. Physiol. **165**, 254–262 (2013). 23507568 10.1016/j.cbpa.2013.03.016

[52] N.-Y. Liu , Two subclasses of odorantd Physiology Part A: Spodoptera exigua display structural conservation and functional divergence. Insect Mol. Biol. **24**, 167–182 (2015). 25345813 10.1111/imb.12143

[53] T.-T. Zhang , Characterization of three pheromone-binding proteins (PBPs) of Helicoverpa armigera (Hübner) and their binding properties. J. Insect Physiol. **58**, 941–948 (2012). 22549127 10.1016/j.jinsphys.2012.04.010

[54] M. C. Larsson , Or83b encodes a broadly expressed odorant receptor essential for *Drosophila* olfaction. Neuron **43**, 703–714 (2004). 15339651 10.1016/j.neuron.2004.08.019

[55] R.-T. Li, L.-Q. Huang, J.-F. Dong, C.-Z. Wang, A moth odorant receptor highly expressed in the ovipositor is involved in detecting host-plant volatiles. eLife **9**, e53706 (2020). 32436842 10.7554/eLife.53706 PMC7308088

[56] M. T. Tom, L. Cortés Llorca, S. Bucks, S. Bisch-Knaden, B. S. Hansson, Sex-and tissue-specific expression of chemosensory receptor genes in a hawkmoth. Front. Ecol. Evol. **10**, 976521 (2022).

[57] N. Fukuda, K. Yomogida, M. Okabe, K. Touhara, Functional characterization of a mouse testicular olfactory receptor and its role in chemosensing and in regulation of sperm motility. J. Cell Sci. **117**, 5835–5845 (2004). 15522887 10.1242/jcs.01507

[58] M. A. Ali , Odorant and taste receptors in sperm chemotaxis and cryopreservation: Roles and implications in sperm capacitation, motility and fertility. Genes **12**, 488 (2021). 33801624 10.3390/genes12040488 PMC8065900

[59] K. R. Ram, M. F. Wolfner, Sustained post-mating response in *Drosophila melanogaster* requires multiple seminal fluid proteins. PLoS Genet. **3**, e238 (2007). 18085830 10.1371/journal.pgen.0030238 PMC2134937

[60] M. K. Manier , Resolving mechanisms of competitive fertilization success in *Drosophila* melanogaster. Science **328**, 354–357 (2010). 20299550 10.1126/science.1187096

[61] B. G. Dias, K. J. Ressler, Parental olfactory experience influences behavior and neural structure in subsequent generations. Nat. Neurosci. **17**, 89–96 (2014). 24292232 10.1038/nn.3594 PMC3923835

[62] Y.-D. Wang , Structural basis for odorant recognition of the insect odorant receptor OR-Orco heterocomplex. Science **384**, 1453–1460 (2024). 38870272 10.1126/science.adn6881

[63] J. Zhao, A. Q. Chen, J. Ryu, J. Del Mármol, Structural basis of odor sensing by insect heteromeric odorant receptors. Science **384**, 1460–1467 (2024). 38870275 10.1126/science.adn6384 PMC11235583

[64] J.-H. Hou , Identification and expression specificity of chemosensory genes in the male reproductive system of *Spodoptera exigua*. J. Asia-Pac. Entomol. **26**, 102097 (2023).

[65] M. A. El-Brolosy, D. Y. R. Stainier, Genetic compensation: A phenomenon in search of mechanisms. PLoS Genet. **13**, e1006780 (2017). 28704371 10.1371/journal.pgen.1006780 PMC5509088

[66] A. L. Barabasi, Z. N. Oltvai, Network biology: Understanding the cell’s functional organization. Nat. Rev. Genet. **5**, 101–113 (2004). 14735121 10.1038/nrg1272

[67] L. Jiang , Compensatory effects of other olfactory genes after CRISPR/cas9 editing of BmOR56 in silkworm, *Bombyx mori*. Comp. Biochem. Physiol. Part D. Genomics Proteomics **52**, 101275 (2024). 38901107 10.1016/j.cbd.2024.101275

[68] P. Kranzfelder, T. Ekrem, E. Stur, Trace DNA from insect skins: A comparison of five extraction protocols and direct PCR on chironomid pupal exuviae. Mol. Ecol. Resour. **16**, 353–363 (2016). 26186122 10.1111/1755-0998.12446

[69] Y.-G. Liu, Y. Chen, High-efficiency thermal asymmetric interlaced PCR for amplification of unknown flanking sequences. BioTechniques **43**, 649–656 (2007). 18072594 10.2144/000112601

[70] Z.-Q. Teng, Q.-W. Zhang, Determinants of male ejaculate investment in the cotton bollworm Helicoverpa armigera: Mating history, female body size and male age. Physiol. Entomol. **34**, 338–344 (2009).

[71] Y. He, Spodoptera exigua Raw sequence reads. NCBI. https://www.ncbi.nlm.nih.gov/bioproject/PRJNA1234362. Deposited 11 March 2025.

