# Supplementary material for: Male-derived PBP4 is essential for sperm competition by mediating sperm motility in moths
Source: Proc Natl Acad Sci U S A. 2025 Oct 28;122(44):e2510155122. doi: 10.1073/pnas.2510155122 (PMC12595504; doi:10.1073/pnas.2510155122)
Supplement: Supplementary file 1 — Appendix 01 (PDF) [file pnas.2510155122.sapp.pdf]

Supporting Information for
Male derived PBP4 is essential for sperm competition by mediating sperm motility in moths

Yu He[1], Qi Yan[1,2], Jing-Hao Hou[1], Ying Li[1], Zhi-Qiang Wei[1], Jin-Meng Guo[1], Nai-Yong Liu[3], Markus Knaden[5], Bill S. Hansson[5], Shuang-Lin Dong[1,2]*, Jin Zhang[1,2,4]*

* Jin Zhang; Shuang-Lin Dong.
Email: jinzhang001@njau.edu.cn; sldong@njau.edu.cn

This PDF file includes:

Figures S1 to S10
Tables S1 to S7
Legends for Movies S1 to S7

Other supporting materials for this manuscript include the following:

Movies S1 to S7

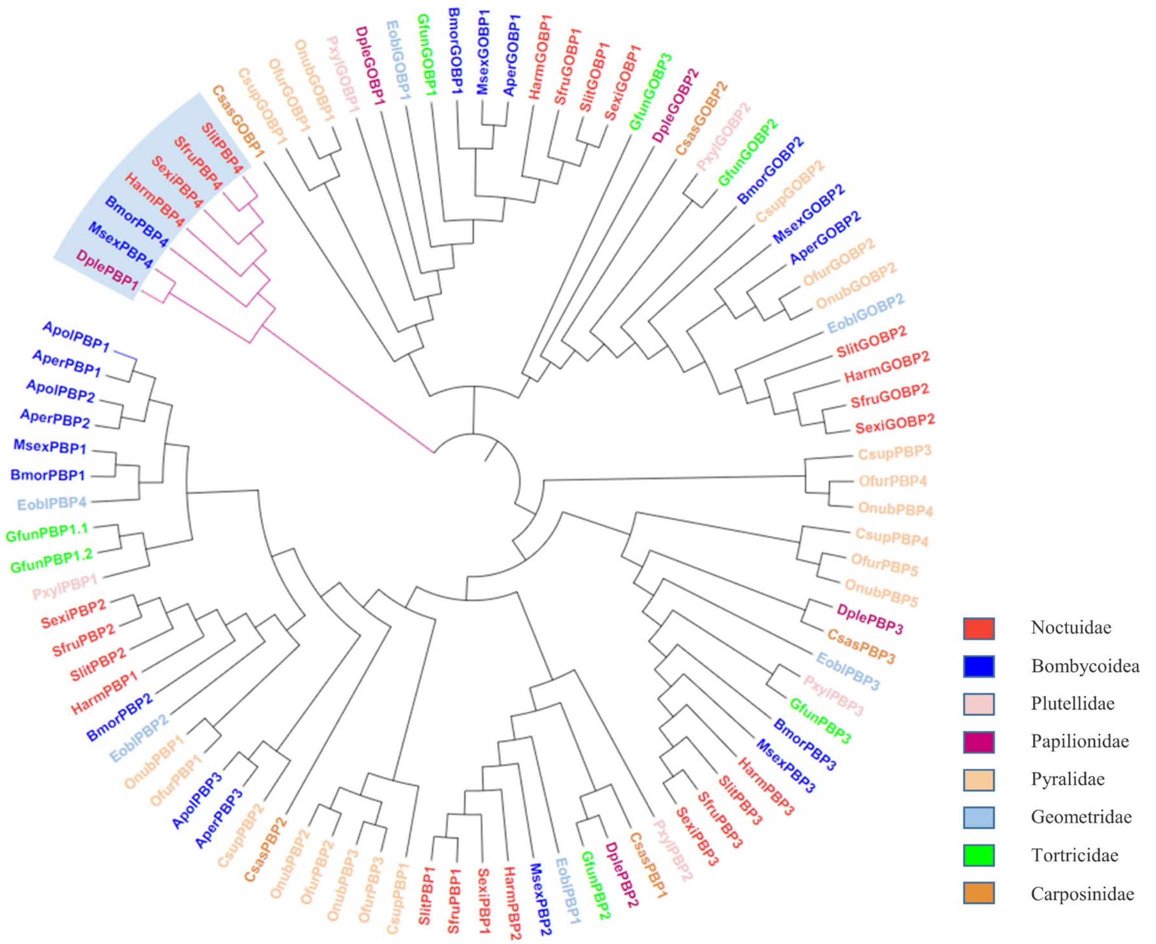

Fig. S1. Phylogenetic analysis of PBPs and GOBPs among 16 species. Sexi: *S. exigua*; Slit: *S. litura*; Sfru: *S. frugiperda*; Harm: *H. armigera*; Csas: *C. sasakii*; Gfun: *G. funebrana*; Eobl: *E. obliqua*; Onub: *O. nubilalis*; Ofur: *O.a furnacalis*; Csup: *C. suppressalis*; Dple: *D. plexippus*; Apol: *A. Polyphemus*; Aper: *A. pernyi*; Msex: *M. sexta*; Bmor: *B. mori*; Pxyl: *P. xylostella*.

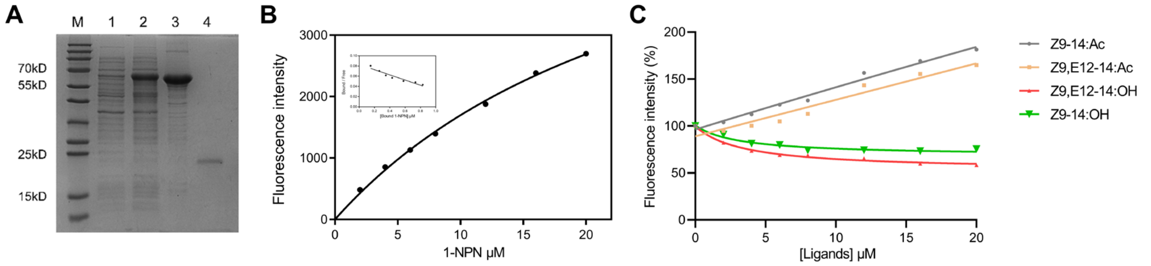

Fig. S2. The binding affinity of recombinant PBP4 protein to four sex pheromones of *S. exigua*. (**A**) Expression of recombinant PBP4 protein. M, Protein maker; Lane 1: The proteins of uninduced bacterial liquid; Lane 2: The proteins of IPTG induced bacterial liquid; Lane 3: The purified recombinant protein; Lane 4: The digested recombinant protein. (**B**) The fitting curve of PBP4 binding to probe 1-NPN. (**C**) Binding curves of PBP4 to four sex pheromones.

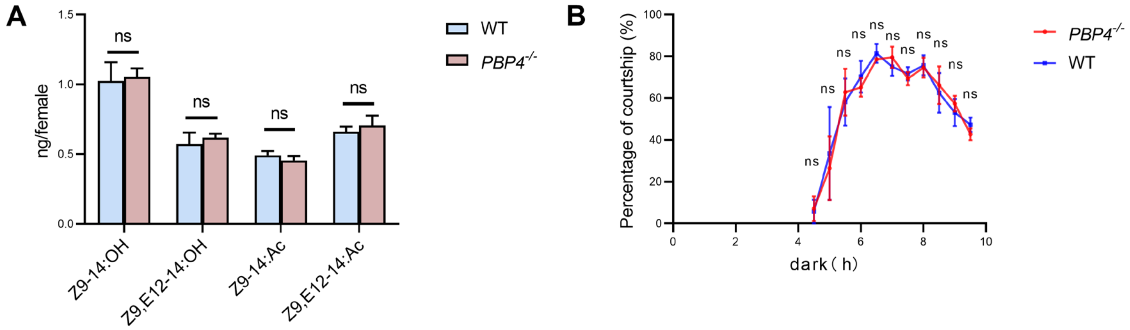

Fig. S3. The pheromone content at 2-3 hours (A) and courtship rhythm on the second day (B) after mating of mates of *PBP4*$^{-/-}$ or WT males. Error bar means SEM (n = 3-4). “ns” indicates no significant difference between WT and *PBP4*$^{-/-}$ females (Student's t-test, $P < 0.05$).

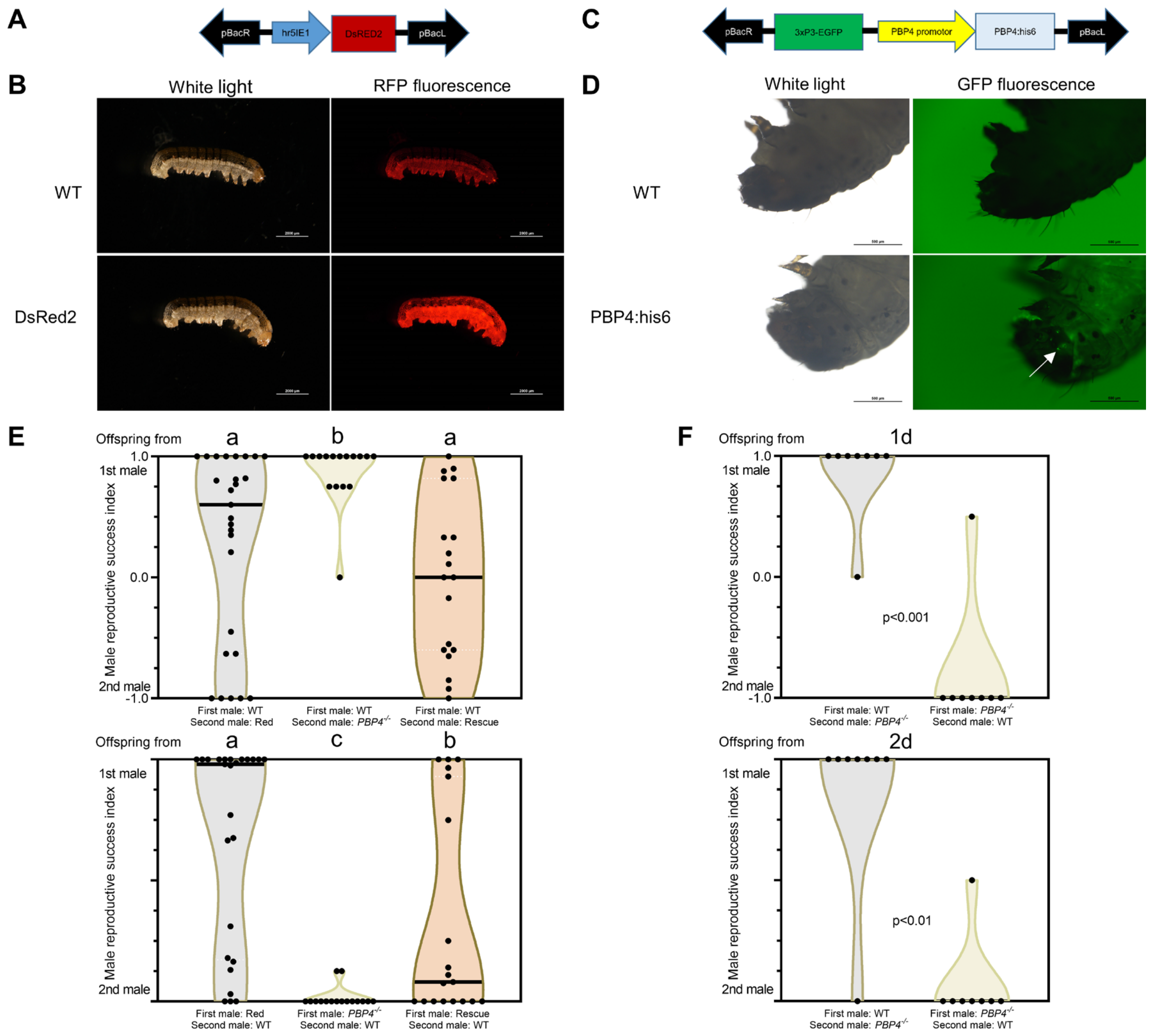

Fig. S4. The construction of transgenetic moth and male sperm competition. (**A**) Diagram of the structure of pBac[DsRed2] vector. (**B**) Bright-field and red fluorescent images of the transgenic larvae. (**C**) Diagram of the structure of pBac[PBP4:his6] vector. (**D**) Bright-field and fluorescent images of the transgenic larvae. White arrow indicates the eyes of larve. (**E**) Violin plots represent male reproductive success index on the second day. Different letters indicate significant differences among groups (Welch and Brown-Forsythe one-way ANOVA with Tamhane T2 test, $P < 0.05$). (**F**) Violin plots represent male reproductive success index after mating with a *PPP4* knockout female. The terms "1d" and "2d" denote the offspring produced on the first and second day, respectively, by females that mated twice. The difference of male reproductive success index after changing the order of mating was tested via Mann-Whitney U test.

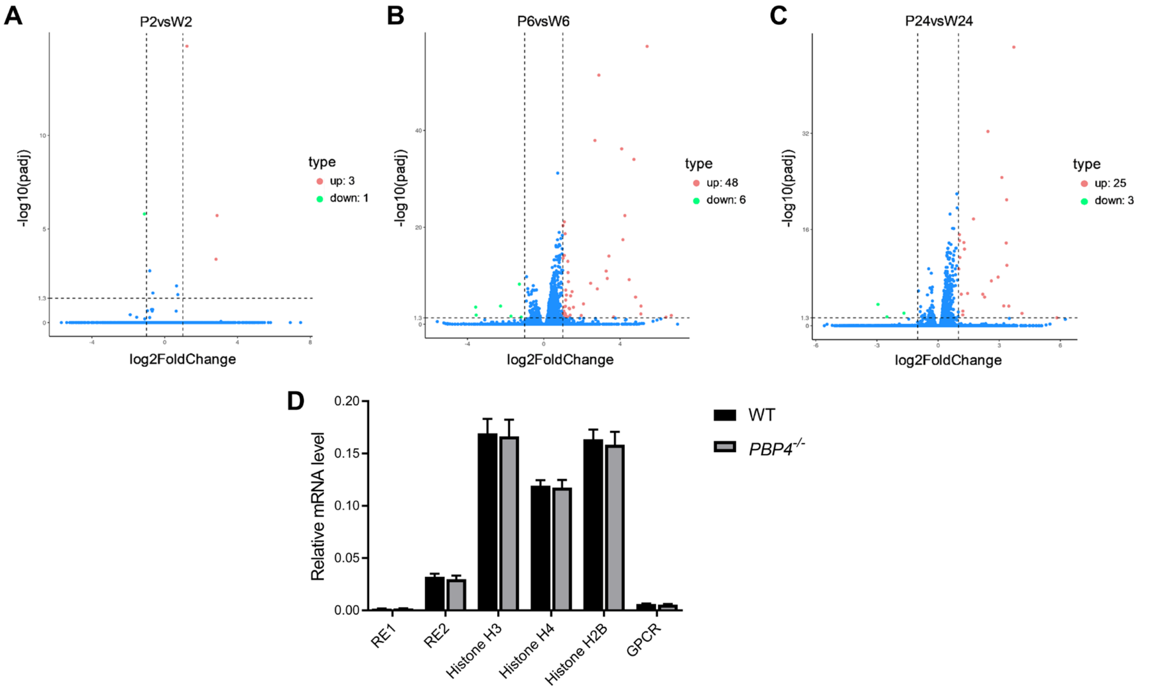

Fig. S5. Volcanic pot of DEGs of abdomen and head of mates of *PBP4$^{-/-}$* and WT males 2 h (A), 6 h (B), 24 h (C) after mating and qRT-PCR analysis (D) of 6 candidate DEGs. RE1, retrotransposable element 1; RE2, retrotransposable element 2; GPR, G protein-coupled receptor. Error bar means SEM (n = 3). "ns" indicates no significant difference between WT and *PBP4$^{-/-}$* females (Student's t-test, $P < 0.05$).

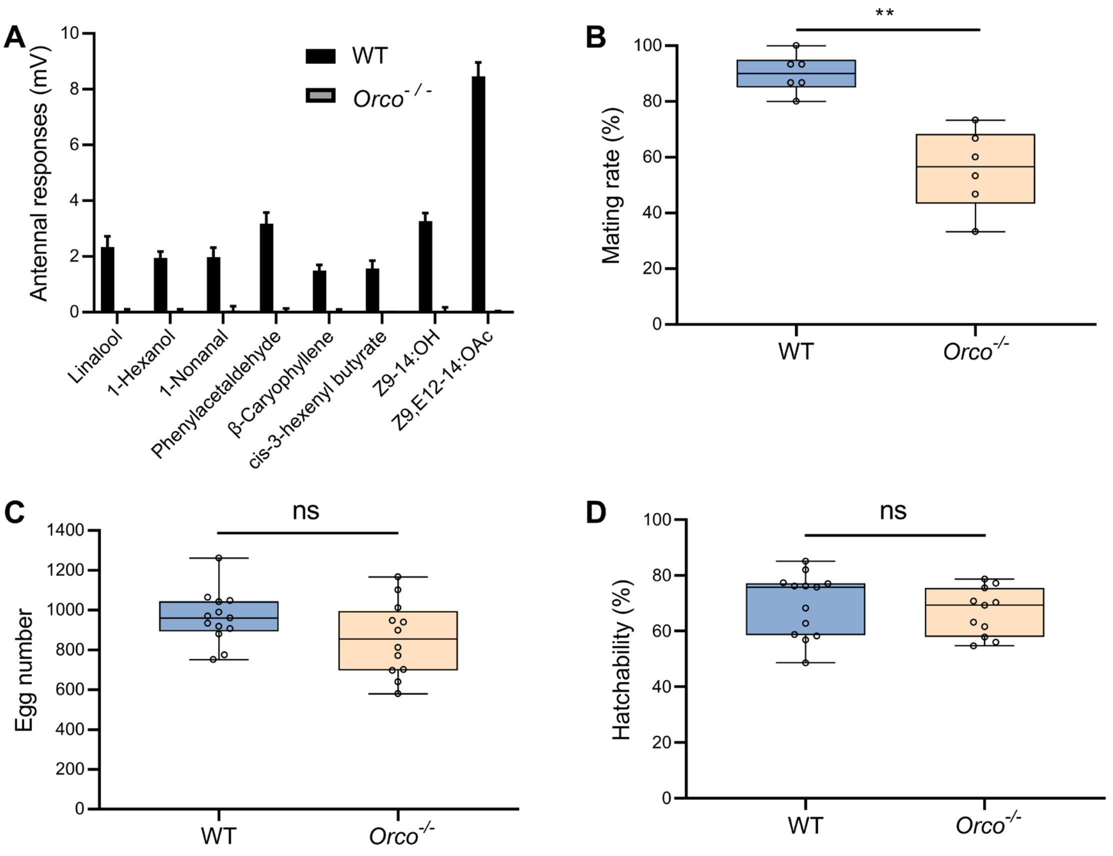

Fig. S6. The EAG responses and reproductive phenotypes of WT and *Orco*$^{-/-}$ strain. (**A**) EAG responses of WT and *Orco*$^{-/-}$ males (n = 10). Error bar represents mean ± SEM. (**B**) Mating rate of mates of WT (n = 6) or *Orco*$^{-/-}$ males (n = 6). (**C**) Egg number of mates of WT (n = 13) or *Orco*$^{-/-}$ males (n = 12). (**D**) Hatchability of mates of WT (n = 13) or *Orco*$^{-/-}$ males (n = 11). Boxplots depict median, upper, and lower quartiles. ** indicate significant difference between different groups (Student's t-test, $**P < 0.01$)(**B**) ; “ns” indicates no significant difference between different groups (Student's t-test, $P < 0.05$)(**C**, **D**).

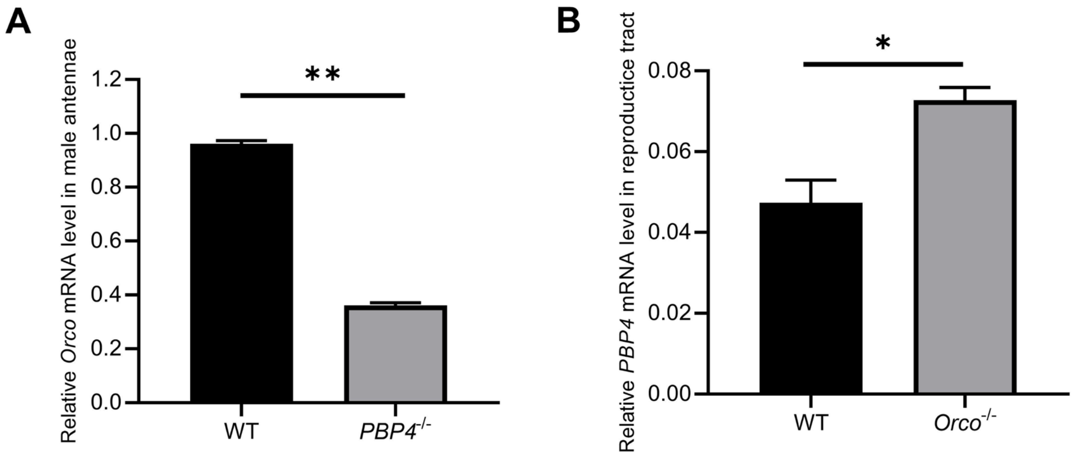

Fig. S7. The *Orco* and *PBP4* genes expression changes in different strains. (**A**) The *Orco* gene expression level in antennae of WT and *PBP4*$^{-/-}$ males. (**B**) The *PBP4* gene expression level in reproductive tracts of WT and *Orco*$^{-/-}$ males. Error bar means SEM (n = 3). * indicate significant difference between different groups (Student's t-test, **P* < 0.05, ***P* < 0.01).

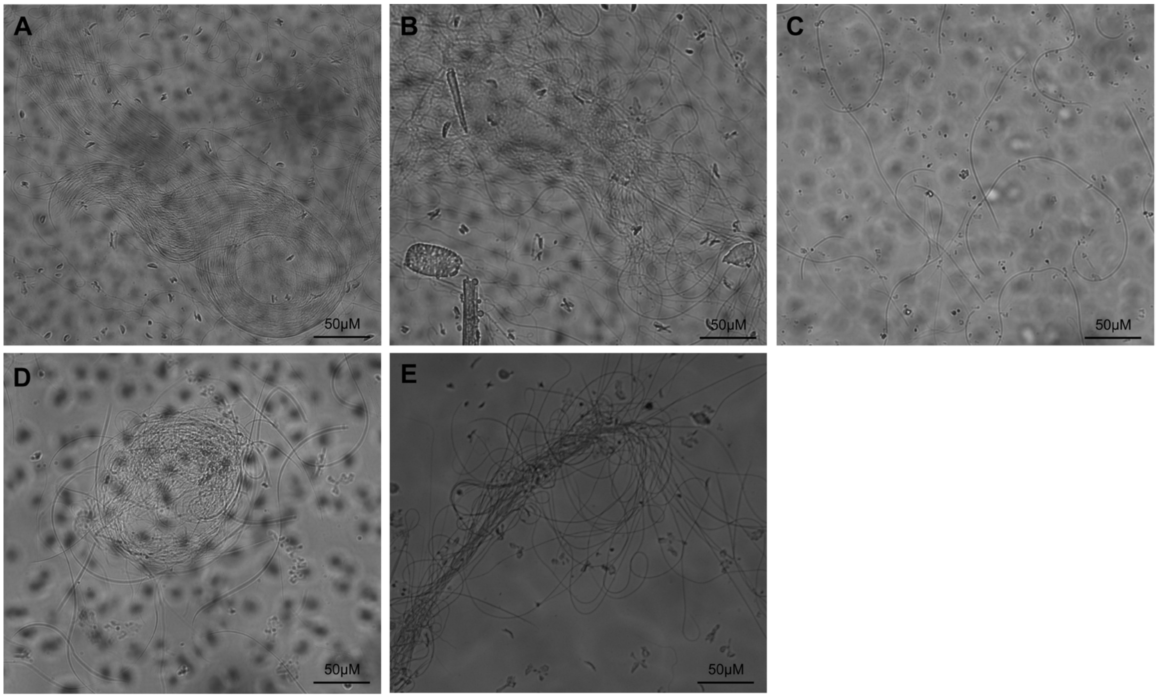

Fig. S8. The different sperm status in the spermatophore and spermatheca. (**A**) Undissociated eupyrene sperm bundle. (**B**) Incompletely dissociated apyrene sperm bundle. (**C**) Free eupyrene sperm. (**D**) Sperm clump. (**E**) Incompletely dissociated sperm bundle.

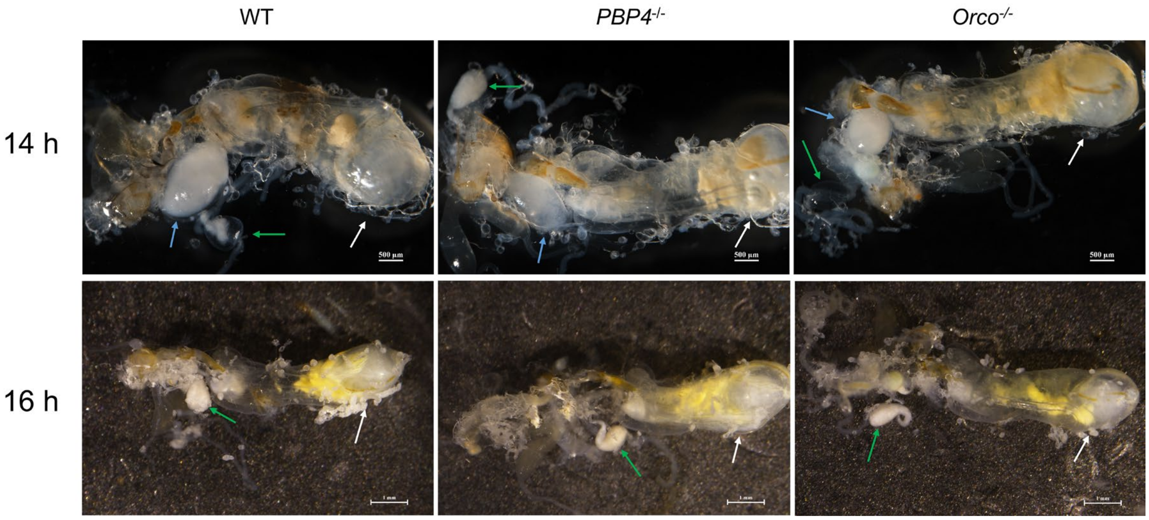

Fig. S9. The reproductive tract of females 14 h and 16 h after mating with males of three strains. White arrow indicates the spermatophore; blue arrow indicates a bursa that appears only during sperm transfer; green arrow indicates the spermathecal.

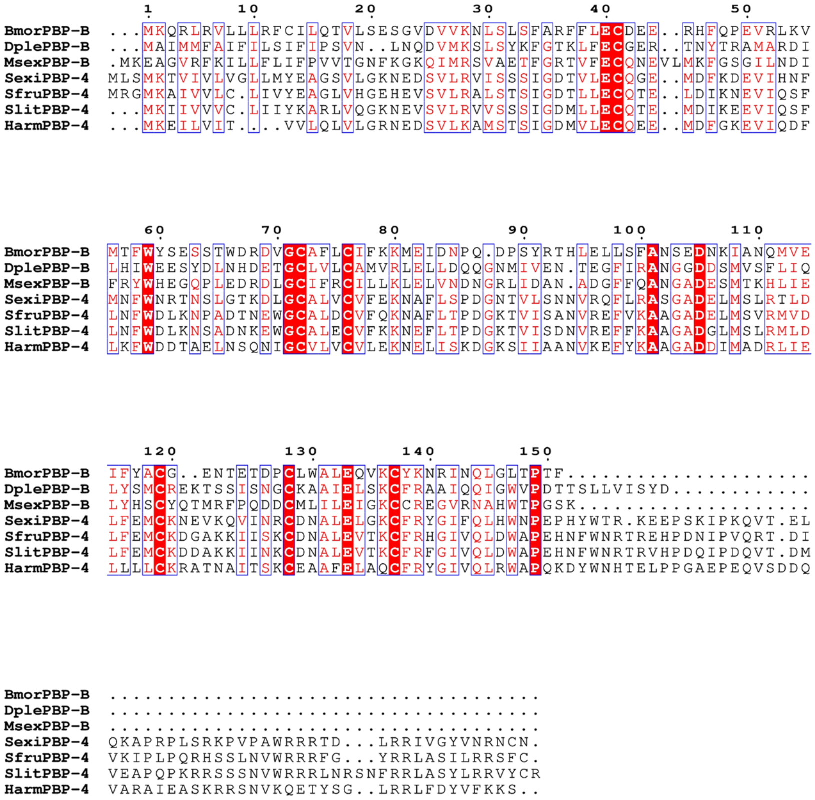

Fig. S10. Sequence analysis between PBP4s of Noctuidae species and PBP-Bs of non-Noctuidae species. Bmor: *B. mori*; Dple: *D. plexippus*; Msex: *M. sexta*; Sexi: *S. exigua*; Sfru: *S. frugiperda*; Slit: *S. litura*; Harm: *H. armigera*.

Table S1. Primers used in this study.

Primers	5'-3' sequence
**For qRT-PCR**	
12223-qF	GCAGTCTTCTCCGTATGA
12223-qR	TGTCTATGGTGGCGTTAG
2608-qF	GGTTGTGGACGGTATAGT
2608-qR	TTACCAGCATAGCGACTC
0-qF	ACCTACAAGCGTGATACC
0-qR	ACCTCGTGAGTATATTAAGTAGT
Orco-qF	CAGCAGAACAACAATCCTAA
Orco-qR	GGTAAGCCAGTAGAGTAAGT
29005-qF	CATTATCGCAGGCTATTGG
29005-qR	GCAGTTGAAGACGATGAC
13141-qF	GGATATGTCTGGGATAGTGAA
13141-qR	TGGTTGTAGTAATCGTAGCA
13710-qF	TGGTTGGAGTGAACAAGT
13710-qR	GATGATGGAAGAGGATGGT
8615-qF	CCGACCGACTTATCTACC
8615-qR	CGACCACCAGAATACTGTA
13996-qF	ATCGGTAGAGGAACACATC
13996-qR	GAAGAGGCAGAGGGTATG
16327-qF	GCCGAACCGTATTTCATC
16327-qR	CAGAGTCTCGCCAATAATG
20711-qF	ACATATCCAACGCCTTCA
20711-qR	CAAGAGTGCCCGTAAATAC
7636-qF	CGTCGTTACCAGAAGAGT
7636-qR	ATCGGTCTTGAAGTCCTG
7635-qF	TACTCCGTGATAACATCCAG
7635-qR	AGGAACACCTTCAACACA
19405-qF	TACATCTACAAGGTGCTGAA
19405-qR	GGTGATGGTCGATCTCTT
GAPDH-qF	GACAACCACTCATCTATCTTCG
GAPDH-qR	AACATTTATCTCTACAACGCAAC
PBP4-qF	TGCTTAGTATGAAGACAGTTA
PBP4-qR	ACGCTATCCTCATTCTTG
**For sgRNA synthesis**	
sgSexiPBP4-F	TAATACGACTCACTATAGCGTGTCATCAGCAGCAGC
sgSexiPBP4-R	TTCTAGCTCTAAAACATGCTGCTGCTGATGACA
sgSlitPBP4-F	TAATACGACTCACTATA**G**TACAAGGCGAGGCTGGTG
sgSlitPBP4-R	TTCTAGCTCTAAAACTGCACCAGCCTCGCCTT
sgOrco-F	TAATACGACTCACTATAGGTAGATCTTACGAAGAAG
sgOrco-R	TTCTAGCTCTAAAACGCCTTCTTCGTAAGATCT
**For *piggyBac* vector construction**	

3xP3-F	GCCCTTGCTCACCATGCGGCCGCGGTGGCGACCGGTGGATC
3xP3-R	GGTACCCAATTCGCCCTATAGGTTCCCACAATGGTTAATTCGAG
hr5IE1-F	CTATAGGGCGAATTGGGTACCCATTGCTTGTCATTTATTAATTTGGA
hr5IE1-R	ACGTTCTCGGAGGAGGCCATTCTTGTCGCCGCCAGTGT
DsRed2-F	ATGGCCTCCTCCGAGAAC
DsRed2-R	TTATGATCTAGAGTCGGGCCCCTACAGGAACAGGTGGTGGCG
PBP4-pro-F	CAGTTCGGGACATAAATGTTTAAATATACTTAAATCACTCAACACCTTTAAACT
PBP4-pro-R	TGCCTTAAACTTCTTAATATTTCCTTCT
PBP4:his6-F	ATATTAAGAAGTTTAAGGCAATGCTTAGTATGAAGACAGTTATTG
PBP4:his6-R1	CAGTGGTGGTGGTGGTGGTGATTACAATTTCTATTAACGTAACCC
PBP4:his6-R2	TTATGATCTAGAGTCGGGCCCTCAGTGGTGGTGGTGGTGGTG
**For semi-quantitative RT-PCR**	
SfruPBP4-F	ATGCTTGGTATGAAGGCAGT
SfruPBP4-R	GCCGTCTGTAACCGAATCG
HarmPBP4-F	ATGAAGGAGATTTTGGTGATAACTG
HarmPBP4-R	TGAAAACGTAATCAAAGAGCCG
BmorPBP4-F	ATGAAGCAACGTCTGAGAGT
BmorPBP4-R	TTAGAACGTCGGAGTCAAACC
SfruGAPDH-F	CTTCATTGGTCTTGACTACATGG
SfruGAPDH-R	GGTTGGAGTAACCGTACTCG
HarmGAPDH-F	ATGTCCAAAATCGGTATCAACG
HarmGAPDH-R	TTAATCCTTGGTCTGGATGTACTT
BmorGAPDH-F	ATGTCAAAAATTGGAATCAATGGATT
BmorGAPDH-R	CATTTGAGATGACCTTAAAAGAGGG

Table S2. The four sex pheromones used for fluorescence competition binding assay.

Reagent	Source	CAS number
Z9-14:Ac	IMROG	16725-53-4
Z9,E12-14:Ac	IMROG	31654-77-0
Z9,E12-14:OH	IMROG	42521-46-0
Z9-14:OH	IMROG	35153-15-2

Table S3. Reproductive phenotypes of mates of *PBP4* knockout or WT males.

Assay	Females mated to WT males (n)	Females mated to knockout males (n)
Egg laying	824.1 ± 26.0(25)	784.6 ± 37.3(23)
Hatchability (%)	74.4 ± 1.6(15)	69.3 ± 5.0(11)
Mating rate (%)	90.8 ± 2.2(8)	86.7 ± 3.3(8)
Remating rate (%)	68.8 ± 3.7(8)	72.8 ± 5.3(8)

Egg laying: Number of eggs laid by a mated female for 4 days after the start of mating (P = 0.38).
Hatchability: Percentage of laid eggs reaching larval stage (P = 0.34).
Mating rate: Ratio of virgin females that mated in a photoperiod (15 pairs every repeat, P = 0.31).
Remating rate: Ratio of gravid females that remated in a photoperiod (15 pairs every repeat, P = 0.54).
Values given are mean ± SE with sample sizes in parentheses. Statistics were performed with Student’s t test.

Table S4. Transgenic insertion genome location information.

Strain	Chromosome	Location	Detail information
DsRed2	Chr18	3500151	between Sexi001134 and Sexi001224
PBP4:his6	Chr20	10438929	between Sexi017785 and Sexi017776

Table S5. Egg laying and hatchability of mates of WT, DsRed2 or PBP4:his6 males.

Assay	Females mated to WT male (n)	Females mated to DsRed2 male (n)	Females mated to PBP4:his6 male (n)
Egg laying	854.1 ± 33.5(14)	807.7 ± 27.3(15)	829.1 ± 15.0(15)
Hatchability (%)	73.8 ± 4.2(14)	77.5 ± 5.6(15)	73.3 ± 9.0(15)

Table S6. Quantitative real-time validation of the transcriptome analysis.

Gene ID	log2Fold change of readcount (PRE vs WRE)	NT Description
**9 DEGs of male reproductive tract**		
Cluster-10283.12223	1.5648	glucose-induced degradation protein
Cluster-10283.2608	2.1423	sorbitol dehydrogenase
Cluster-17444.0	1.4216	glucose dehydrogenase
Cluster-10283.5806	7.3808	odorant receptor coreceptor
Cluster-10283.29005	2.6388	dynein heavy chain
Cluster-10283.13141	6.4587	zonadhesin
Cluster-10283.13710	-1.0891	ATP-citrate synthase
Cluster-10283.8615	-1.6387	major royal jelly protein
Cluster-10283.13996	-1.0275	ATP-binding cassette
**6 DEGs of female abdomen and head**		
Cluster-10283.16327	4.7274	retrotransposable element 1
Cluster-10283.20711	4.0871	retrotransposable element 2
Cluster-10283.7636	1.1797	Histone H3
Cluster-10283.7635	1.1515	Histone H4
Cluster-10283.19405	1.1355	Histone H2B
Cluster-10283.7164	1.1206	G protein-coupled receptor

Table S7. Compounds used for electroantennogram recording.

Reagent	Source	CAS number
Linalool	Macklin	78-70-6
1-Hexanol	Macklin	111-27-3
1-Nonanal	Macklin	124-19-6
Phenylacetaldehyde	Macklin	122-78-1
β-Caryophyllene	Macklin	87-44-5
cis-3-hexenyl acetate	Macklin	3681-71-8
Z9-14:OH	Nimord Changzhou	16725-53-4
Z9,E12-14:OAc	Nimord Changzhou	30507-70-1

Movie S1 (separate file). The depiction of different motility grades for apyrene sperm.

Movie S2 (separate file). The apyrene sperm motility in WT males.

Movie S3 (separate file). The apyrene sperm motility in WT males after addition of 10^{-4} M VUAA1.

Movie S4 (separate file). The apyrene sperm motility in *PBP4*$^{-/-}$ males.

Movie S5 (separate file). The apyrene sperm motility in *PBP4*$^{-/-}$ males after addition of 10^{-4} M VUAA1.

Movie S6 (separate file). The apyrene sperm motility in *Orco*$^{-/-}$ males.

Movie S7 (separate file). The apyrene sperm motility in *Orco*$^{-/-}$ males after addition of 10^{-4} M VUAA1.
